# Analytical methods in studying cell force sensing: principles, current technologies and perspectives

**DOI:** 10.1093/rb/rbaf007

**Published:** 2025-03-20

**Authors:** Xiaojun Liu, Lei Yu, Adam Xiao, Wenxu Sun, Han Wang, Xiangxiu Wang, Yanghao Zhou, Chao Li, Jiangtao Li, Yongliang Wang, Guixue Wang

**Affiliations:** College of Life Sciences and Health, University of Health and Rehabilitation Sciences, Qingdao 266113, China; Qingdao Municipal Hospital, University of Health and Rehabilitation Sciences, Qingdao 266024, China; Department of Traditional Chinese Medicine, Qingdao Special Service Sanatorium of PLA Navy, Qingdao 266071, China; Department of Chemistry, University of British Columbia, Vancouver, BC V6T 1Z1, Canada; School of Sciences, Nantong University, Nantong 226019, China; State Key Laboratory of Precision Measuring Technology and Instruments, School of Precision Instrument and Optoelectronics Engineering, Tianjin University, Tianjin 300072, China; Key Laboratory for Biorheological Science and Technology of Ministry of Education, State and Local Joint Engineering Laboratory for Vascular Implants, Bioengineering College of Chongqing University, Chongqing 400030, China; Key Laboratory for Biorheological Science and Technology of Ministry of Education, State and Local Joint Engineering Laboratory for Vascular Implants, Bioengineering College of Chongqing University, Chongqing 400030, China; College of Life Sciences and Health, University of Health and Rehabilitation Sciences, Qingdao 266113, China; Qingdao Municipal Hospital, University of Health and Rehabilitation Sciences, Qingdao 266024, China; College of Life Sciences and Health, University of Health and Rehabilitation Sciences, Qingdao 266113, China; College of Life Sciences and Health, University of Health and Rehabilitation Sciences, Qingdao 266113, China; Qingdao Municipal Hospital, University of Health and Rehabilitation Sciences, Qingdao 266024, China; Key Laboratory for Biorheological Science and Technology of Ministry of Education, State and Local Joint Engineering Laboratory for Vascular Implants, Bioengineering College of Chongqing University, Chongqing 400030, China; Qindao Central Hospital, University of Health and Rehabilitation Sciences, Qingdao 266044, China; Key Laboratory for Biorheological Science and Technology of Ministry of Education, State and Local Joint Engineering Laboratory for Vascular Implants, Bioengineering College of Chongqing University, Chongqing 400030, China; JinFeng Laboratory, Chongqing 401329, China

**Keywords:** biomaterial–cell interaction, biosensor, cell biomechanics, biomaterial design

## Abstract

Mechanical stimulation plays a crucial role in numerous biological activities, including tissue development, regeneration and remodeling. Understanding how cells respond to their mechanical microenvironment is vital for investigating mechanotransduction with adequate spatial and temporal resolution. Cell force sensing—also known as mechanosensation or mechanotransduction—involves force transmission through the cytoskeleton and mechanochemical signaling. Insights into cell–extracellular matrix interactions and mechanotransduction are particularly relevant for guiding biomaterial design in tissue engineering. To establish a foundation for mechanical biomedicine, this review will provide a comprehensive overview of cell mechanotransduction mechanisms, including the structural components essential for effective mechanical responses, such as cytoskeletal elements, force-sensitive ion channels, membrane receptors and key signaling pathways. It will also discuss the clutch model in force transmission, the role of mechanotransduction in both physiology and pathological contexts, and biomechanics and biomaterial design. Additionally, we outline analytical approaches for characterizing forces at cellular and subcellular levels, discussing the advantages and limitations of each method to aid researchers in selecting appropriate techniques. Finally, we summarize recent advancements in cell force sensing and identify key challenges for future research. Overall, this review should contribute to biomedical engineering by supporting the design of biomaterials that integrate precise mechanical information.

## Graphical abstract

**Figure:**
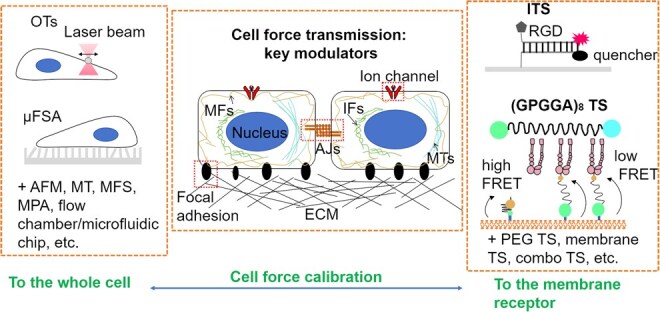

## Introduction

Mammalian cells adhere to the extracellular matrix (ECM) through integrins, which serve as the primary adhesion receptors connecting to the cytoskeleton, including microfilaments and microtubules. Mechanical forces propagate along the cytoskeleton, leading to the elongation of mechanosensors such as talin and vinculin. These changes first affect the cytoskeleton and subsequently influence the nuclear skeleton. Mechanosensation transforms mechanical stimuli into electrical or chemical signals, interpreted through ion fluxes and the activation of protein kinases and downstream biochemical signaling. Typically, force transduction along microfilaments involves piconewton (pN) scales, while single ion currents range from several picoamperes, necessitating advanced measurement techniques. This review begins by outlining the structural basis of cell force sensing, relevant signaling pathways, the clutch model and the role of mechanotransduction in both physiological and pathological contexts, as well as its implications for mechanical biomaterial design. The second part focuses on various methods for measuring cell forces, with an emphasis on fluorescent force sensors, and concludes with a perspective on the field’s future directions.

## The structural components in cell force sensing

**Figure 1.**
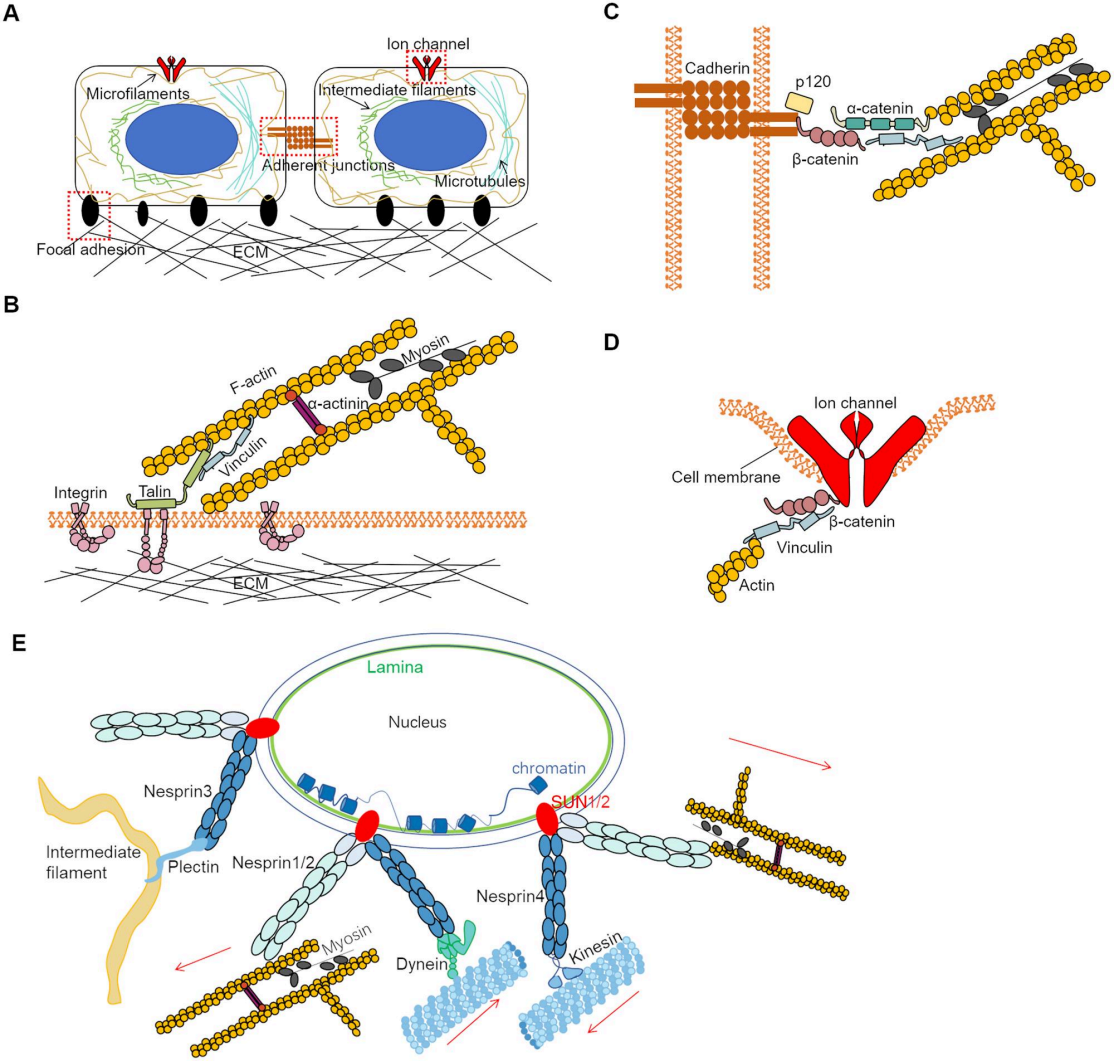
The cell cytoskeleton and force-sensitive machinery. (**A**) Two cells adhered to the extracellular matrix (ECM) through focal adhesions and adherent junctions via cadherins, with mechanosensitive ion channels on the membrane. Microfilaments, microtubules and intermediate filaments work together to support cell shape alternations under various conditions. (**B**) Portrayal of the focal adhesion and acto-myosin system. Herein, adaptor proteins talin and vinculin act as force sensors in cell force transmission [9, 18]. Myosin mediates the contraction between actin filaments and the forces propagate toward integrin by talin and vinculin. Only relative higher tension pulling on integrin induces the cluster formation of integrin. (**C**) Cadherins link to MFs via catenins (the adaptor proteins), and then connect to MFs [19, 20]. Similar to focal adhesions, the cell junctions sense forces from the intracellular cytoskeleton. Myosin pulls actin filaments and MFs thereby stretching talin and α-, β-catenin. (**D**) Piezo 1 is a tension-sensitive channel, which is opened by membrane stretching and actomyosin traction force [21–23]. (**E**) Nesprins and SUN1/2 link the nuclear interior to cytoskeletal filaments. Molecular motors interact with these filaments to generate forces, which are transmitted to the nucleus via the linker of nucleoskeleton and cytoskeleton complex (LINC). Genomic regions associated with the lamina, known as lamina-associated chromatin domains (LADs), exhibit low transcriptional activity.

The cell membrane and its associated proteins play key roles in mechanical signal sensing (Figure 1). Integrins, a family of adhesive receptors, mediate cell adhesion and initiate focal complex assembly. These adhesion spots transduce mechanical signals through the cytoskeleton, including microfilaments, microtubules and intermediate filaments, either directly or indirectly. Focal adhesion is tightly regulated; for example, the Arg–Gly–Asp (RGD) nanospacing of integrin ligands subtly influences cell migration [1–3].

The force in cell adhesion or migration travels through a chain or a network, the ECM–integrin–adaptor protein–actin (Figure 1B), comprised hundreds of components all responsible for mechanoregulation. Such complexity also extends to the various types of proteins involved in force transmission, which contains the adaptor proteins talin, filamin [4], tensin [5], α-actinin [6] and kindling [7]. The connection between integrin and actin is mediated by vinculin [8–11], kidney or KN motif and ankyrin repeat domain-containing (kank) [12], focal adhesion kinase [13], paxillin [14]. Myosin motors connect actin fibers and transduce force via contraction within the F-actin network, which develops into the actomyosin system. In addition to energy dissipation, vinculin and talin act as force sensors, facilitating effective regulation of force transmission. This mechanism has been modeled previously, with the ‘molecular clutch’ model being a prominent example [15].

Cell–cell adherent junctions also function as force-responsive machinery. Cadherin–cadherin interactions form dimers in either *cis* or *trans* configurations, mediating force transmission through α- and β-catenin to the actin cytoskeleton [16, 17]. This creates another force chain: cadherin–cadherin–adaptor proteins–actin (Figure 1C). Alpha-catenin binds to F-actin, linking the adhesion site to the cytoskeletal mesh [24, 25], while vinculin serves as an adaptor between F-actin and α-catenin [26]. Beyond this, numerous auxiliary proteins are recruited to the cell–cell junction, as well as in adhesion complexes, in a force-dependent manner (e.g. RhoGEF114, vasodilator-stimulated phosphoprotein, testin (LIN-11, Isl-1, and MEC-3) LIM domain protein and Zyxin) [27, 28].

The adhesive structures mediated by cadherins and integrins connect cells, while the cell membrane can sense osmotic pressure or stretching, influencing the opening/closing of mechanosensitive ion channels such as Piezo and transient receptor potential ion channels TRPC6/TRPA1 channels [21, 29]. Piezo channels play roles in processing pressure, touch, hearing and stretch signals [30] and are enriched in focal adhesions [31]. The Piezo channels will change conformation under lateral tension across the cell membrane [22] (Figure 1D). High-speed atomic force microscopy (AFM) imaging has revealed the reversible ring expansion under applied force, with a measured maximum ring (Piezo 1 channel) occurring at 55pN direct pressure [22]. TRPC6 is involved in regulating vasoconstriction, activating in response to shear stress and cell swelling [32], and can also be activated by G-protein-coupled receptor signaling through ligand binding [33].

In addition to force sensing by cell membrane proteins, nuclear mechanotransduction has emerged as a key focus in cell biomechanics. Structurally, the nuclear transmembrane protein nesprin connects to the cytoskeleton through kinesin (to microtubules), dynein (to microtubules) and plectin (to intermediate filaments), while also directly linking to microfilaments [34] (Figure 1E). Nesprin binds to SUN1/2, inner nuclear membrane (INM) proteins that in turn bind to the nuclear lamina. Together, nesprin, the outer nuclear membrane, INM and SUN1/2 proteins form the linker of nucleoskeleton and cytoskeleton complex [35]. The lamina, underlying the INM, is primarily composed of lamins that interact with other nuclear proteins, chromatin and transcription regulators [36]. As a result, the lamina plays a crucial role in nuclear mechanosensing, gene expression and nuclear shape. Perinuclear actin stress fibers and actomyosin contractility apply compressive forces directly to the nucleus. However, the nucleus has developed mechanisms to adapt to varying cellular conditions; for example, histone H3 lysine 9 trimethylation (H3K9me3) of heterochromatin induces nuclear stiffness and membrane tension, helping to shield DNA from mechanical forces [37]. Furthermore, Piezo-1, located on the endoplasmic reticulum (ER) and connected to the nuclear membrane, responds to nuclear mechanosensing [35].

## The signaling related to cell force sensing

**Figure 2.**
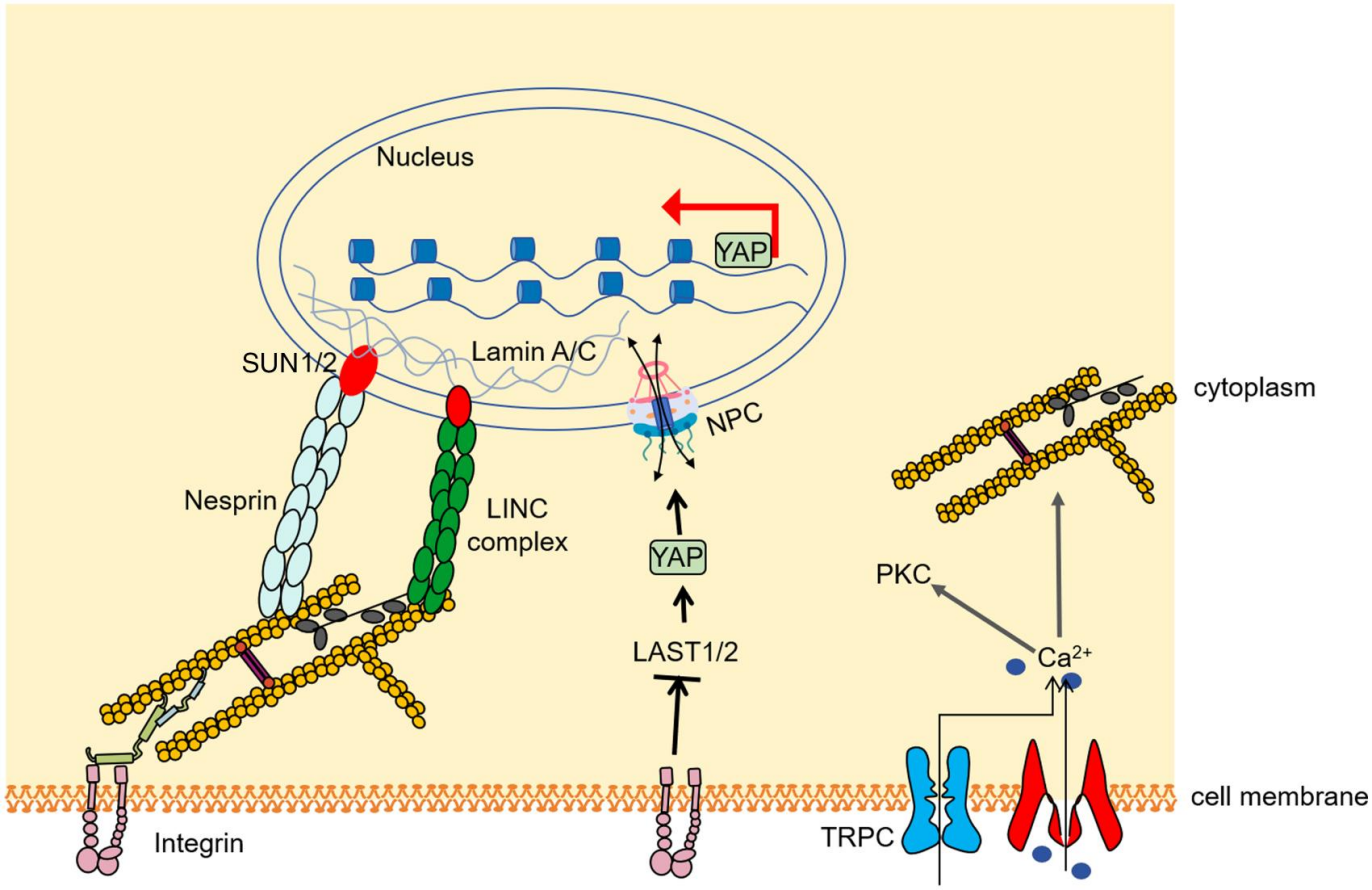
Cell mechanical signaling pathways. Here, three examples are shown including integrin-actomyosin-LINC, integrin-YAP, ion channel-Calcium-kinase and contraction [38, 39, 41–43]. Force transmitted along the integrin-actomyosin-LINC directly induces target gene expression. The force drives YAP translocation into the nucleus and thus modulates gene transcription. Mechanosensitive ion channels such as PIEZO-1 will open upon sensing membrane tension, and increased cytosolic calcium mediates downstream signaling. LINC, linker of nucleoskeleton and cytoskeleton complex; NPC, nuclear pore complex; YAP, Yes-associated protein.

Electrical, mechanical and chemical signals are extensively investigated signaling pathways for cell and molecular biology. Mechanical signals can directly and indirectly influence cell activities (Figure 2). For example, mechanical force may unfold talin, exposing its binding domain for vinculin [38]. This force signal can then be transmitted to the nucleus via the linker of the nucleoskeleton and cytoskeleton complex, regulating gene expression [39]. Also, the mechanochemical coupling enables a broad spectrum of cell reactions. The mechanosensitive ion channels allow transient Ca^2+^ influx as a second signaling messenger, and Ca^2+^ concentration modulates the actomyosin interaction, cell proliferation and differentiation. Calcium levels also affect focal adhesion dynamics, particularly turnover, by influencing the activity of Ca^2+^-sensitive proteins like calpain [40].

As shown in Figure 2, upon experiencing mechanical signals, the dephosphorylation of Yes-associated protein (YAP), a coactivator of gene transcription, at different residues guides its nuclear translocation and modulates gene transcription [41–43]. Generally, direct force transduction appears to act on much faster timescales than that of just chemical signaling.

### The clutch model in force transmission

**Figure 3.**
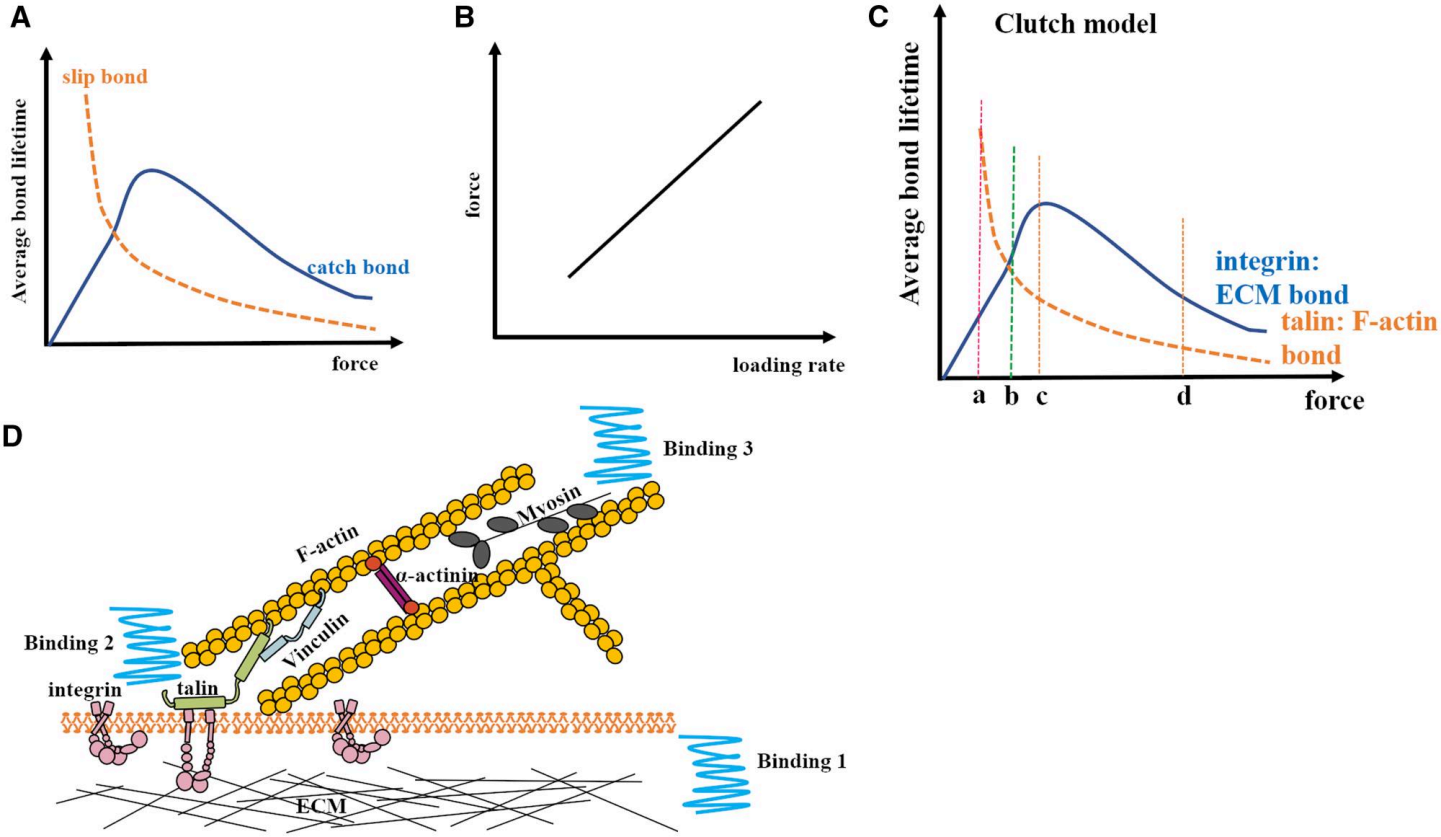
The clutch model. (**A**) The relationship between force and average bond lifetime of slip bond and catch bond [44, 45]. (**B**) The relationship between the force loading rate and force in the corresponded bonding [44]. (**C**) The changes in the lifetime of integrin–ECM and talin–(F-actin) as the force increases [48–52]. (a) The force is small, which is conducive to the formation of talin–(F-actin) bond, and the lifetime is the maximum value. However, the integrin–ECM bond is easy to dissociate, and the integrin activation ratio is very low, making it difficult to pass down to talin and F-actin. (b) Right of the focus of the two curves. Here is the optimal force transduction threshold. When the lifetimes of integrin–ECM bond and talin–(F-actin) bond match, the force transduction process of ECM–integrin–talin–(F-actin) is the smoothest and stable. (c) This is the optimal force of the integrin–ECM bond, but the stability of talin–(F-actin) here has dropped a lot, and the probability that the cell–ECM can form a stable structure is lower than that at b. (d) If the force is too large, the stability of both bonds will be reduced, which is not conducive to cell–ECM connection and force conduction. (**D**) Briefly describes the three binding events that systemically determines the force chain transduction and may work as one clutch or two. The clutch cycle largely depends on the binding lifetime, force level, affected by force loading rate and electrostatic interaction at binding domains [48–52]. (Binding 1: integrin to ECM; binding 2: talin/vinculin to F-actin; binding 3: myosin to F-actin.)

Cell movement is regulated by the actomyosin system and force transduction to the ECM and neighboring cells. Myosin-powered contractility and actin polymerization generate a continuous actin flow, known as retrograde flow, which pushes against the membrane. This flow is only partially transmitted to adaptor proteins and integrins, causing a gradual reduction in retrograde speed as molecules approach the ECM. This reduction in flow suggests a dynamic formation and release of bonds between different molecular elements, which only transmit movement and force when engaged. The working pattern has been previously modeled, with the mechanosensitive molecular clutch model appearing suitable to explain cellular activities in most cases (Figure 3) [15]. The force transmission chain comprises actin–adaptor protein–integrin–ECM or actin–adaptor protein–cadherin–cadherin [15]. Two key parameters to consider are bond force level and bond force lifetime, which are essential for calculating the bond loading rate. These factors describe the affinity and bonding properties between linker proteins in the clutch chain [44]. One type of bond has a longer lifetime upon increasing the force level until reaching the breaking point, named catch bond [45]. The other one shows the decreased lifetime upon increasing the force level monotonously, called slip bond [46] (Figure 3A). Research indicates that talin-F-actin forms a slip bond [47], while integrin–ECM, vinculin–F-actin and myosin–F-actin all develop catch bonds [48–52]. Therefore, force transmission along the molecular chain operates under optimal conditions to achieve maximum force transduction efficiency. We presented a case to explain how the bond association and dissociation in the force transmission along ECM to cytoskeleton (Figure 3C and D). In this context, tension along the molecular chain can be disrupted during unbinding along the force axis and reestablished during binding. This process is influenced by the force level, force loading rate and specific linkages or joints (Figure 3D). The clutch model could explain how the cell fulfills its duty by integrating cell contractility [8], matrix rigidity [8], ECM nanotopography [53] and matrix ligands details (the density, nature and distribution) [54–56] and by controlling the force loading rate in relevant molecules.

### Cell mechanotransduction in physiology and pathology

Mechanical stimulation plays a crucial role in numerous biological activities, including tissue development, regeneration and remodeling [57]. Physiological mechanical signals support normal organ development and repair, whereas prolonged pathological mechanical stimulation leads to irreversible damage. During embryonic development, mechanical signals guide cell differentiation, migration and organization, shaping tissues and organs [58]. For instance, mechanical loading in bone stimulates osteoblast activity and bone formation [59], maintaining bone density and strength. Mechanotransduction is also vital in wound healing, where mechanical signals promote re-epithelialization and tissue remodeling [60]. Similarly, in nerve regeneration, mechanical cues influence axonal growth, facilitating nerve repair and functional recovery [61]. Stretching of cardiomyocytes during each heartbeat triggers biochemical pathways that support heart contractility and growth [62]. However, persistent pathological mechanical stimulation can cause fibrosis, characterized by excessive ECM deposition and tissue stiffening [63]. In the kidneys, stiffened ECM mechanical signals can induce myofibroblast transformation through Wnt and Hh signaling and activate tranforming growth factor (TGF)-induced macrophage cell (M2) polarization by Wnt/β-catenin signaling pathway, promoting renal fibrosis [64, 65]. In the tumor microenvironment, abnormal mechanical properties, such as increased stiffness, influence cancer cell behavior, contributing to tumor growth, metastasis and resistance to therapies [66, 67]. Additionally, abnormal mechanical forces from hypertension can lead to maladaptive cardiac remodeling [68], and in adipose tissue, ECM-associated mechanical cues can trigger system-wide lipotoxicity and insulin resistance [69]. In hyperglycemic conditions, the stiffness of human umbilical vein endothelial cells (ECs) is markedly influenced by glucose concentration, which, in turn, impacts their migration and proliferation capabilities [70].

### Biomechanics and biomaterial design

Cell mechanotransduction is strongly influenced by interactions between membrane receptors and the ECM. Given that the ECM can be characterized by properties such as porosity, elasticity/stiffness, topography and geometry, understanding the effects of each factor on cell behavior is essential. This knowledge can then guide the design of biomaterials optimized for tissue engineering and regenerative medicine. Integrating biomechanics with biomaterials merges the principles of mechanical forces and biological interactions, enabling the development of biomaterials tailored for specific biomedical applications, including tissue engineering, regenerative medicine and prosthetic devices, etc. Here are several ways in which biomechanics and biomaterials can be integrated.

#### Biomechanics-driven material design

Biomaterials can be engineered to replicate the mechanical properties of native tissues, such as stiffness, elasticity and viscoelasticity. For example, in cartilage regeneration, biomaterials with compressive properties similar to native tissue are essential for proper function. It has been shown that stiffer ECMs can promote mesenchymal stem cell (MSC) differentiation toward bone cells [71]. Furthermore, understanding how materials behave under different mechanical loads (tensile, compressive and shear) is crucial for designing materials that can withstand the physiological forces exerted on the body. For instance, the stent strut profile and its response to blood shear stress have provided valuable insights for improving future stent designs [72].

Smart materials have found increasing applications in biomedical engineering. Some biomaterials are designed to alter their mechanical properties in response to external stimuli, such as temperature changes, light exposure, ionic strength, mechanical stress, magnetic fields or pH [73]. This responsiveness is particularly useful in applications like drug delivery systems, where materials can react to the mechanical environment in the body to release therapeutic agents. Another example includes piezoelectric materials, which generate electrical signals in response to mechanical deformation (the piezoelectric effect). These materials can be used to develop devices that sense and respond to biomechanical forces, such as in bone healing or nerve stimulation [74, 75]. 3D bioprinting technique prints structures with varying stiffness and can mimic the natural gradients found in tissues like cartilage or bone [76–78].

#### Design of load-bearing structures

In orthopedic implants, metals such as titanium and its alloys, along with stainless steel and its alloys, are commonly used due to their excellent mechanical strength, toughness and durability. However, polymeric implants [79–81], including poly(lactic acid), poly(glycolic acid), poly(lactide-co-glycolide) and poly(ε-caprolactone), have emerged as alternatives, offering high biocompatibility, non-toxicity and biodegradability. To make these materials viable, it is essential to control their absorption rates and explore methods to enhance their mechanical properties. When designing orthopedic implants, it is also crucial to balance mechanical strength with the material’s ability to integrate with bone or other tissues to prevent stress shielding, where the implant bears most of the load, leading to potential bone resorption.

Additionally, the use of 3D bioprinting allows for the fabrication of structures with varying stiffness, which can replicate the natural gradients found in tissues like cartilage and bone [76–78].

#### Hydrogel for wound dressing

Wound dressing has become a critical area of biomedical materials research. However, treating wounds in challenging areas such as joints, the popliteal fossae, axillae and muscle folds remains difficult. Hydrogels, a class of 3D network gels formed through chemical and/or physical crosslinking, have shown great potential in wound care [82]. A new class of highly stretchable, adhesive, biocompatible and antibacterial hydrogel dressings has been developed by incorporating poly(diallyl dimethyl ammonium chloride) brushes grafted onto bacterial cellulose nanofibers (BC-g-pDADMAC, bBCD) into polydopamine/polyacrylamide hydrogels [83]. These dressings have been shown to provide stable coverage with minimal displacement, long-lasting antibacterial properties and promote rapid wound healing.

#### Cell mechanotransduction and tissue engineering and regeneration

Scaffold design and biomechanical stimulation of cells are critical components in tissue engineering and regeneration projects. The mechanical properties of scaffolds, such as porosity and rigidity, play a key role in influencing cell adhesion, migration and differentiation. Extensive research has highlighted the importance of mechanical cues in modulating cell behavior on various surfaces and within different matrices. In tissue engineering, scaffold porosity significantly impacts mechanical strain, cell density and, consequently, regenerative outcomes [84]. Scaffolds with increased porosity, while maintaining a fixed pore size, have been shown to enhance the proliferation, migration and osteogenic differentiation of skeletal stem and progenitor cells [85]. Furthermore, surface topography cues can be categorized by structure, including roughness coatings, anisotropic patterns (such as grooves and aligned fibers) [86] and isotropic patterns (such as pillars, islands, pits, tubes, columns and fibers). For instance, MSCs cultured on hydroxyapatite discs with roughness levels between 0.2 and 1.65 μm exhibited optimal osteogenic differentiation at a roughness of 0.7–1 μm [87]. Matrix stiffness significantly influences various cell behaviors, including adhesion, proliferation and differentiation. Nuclear transcription factors, such as YAP/TAZ, can be regulated in both activity and localization by changes in matrix stiffness [88]. Additionally, physical geometry impacts cell fate [89]; for example, increased aspect ratios in rectangular shapes or heightened curvature in pentagonal symmetry have been shown to promote osteogenesis in MSCs while suppressing adipogenesis [90].

These insights into cell–matrix interactions provide valuable direction for developing next-generation biomaterials for tissue regeneration. As mechanotransduction data accumulates across different biomaterials, combining physical and chemical signals to control cell fate becomes increasingly feasible. In mechanobiology, a critical challenge lies in measuring cell mechanics with high spatial and temporal precision and in understanding how mechanical cues integrate with biochemical signaling. Consequently, several advanced technologies, detailed in the following section, have been developed to explore how cells respond to their mechanical environment.

## Analytical methods used in cell mechanical studies

In order to measure an invisible force, one has to convert it to a detectable signal. Modern analytical processes utilize various readouts, including electrical (potential, current), chemical and optical (reflection, refraction) signals. Amplifying previously undetectable signals to an analytical level can be achieved through a series of amplifiers, magnification via microscopy and advanced computing resources for signal processing. For details on the fabrication of these instruments, refer to textbooks or manufacturer guides. The following Table 1 summarizes analytical techniques applied in studying force sensing along with their key parameters and major parts.

**Table 1.** The key parameters and major components in the analytical instruments

	Key analytical parameters	Major parts in device	References
TFM (traction force microscope)	Displacement of beads	Microscope; complementary metal oxide semiconductor/charge coupled device (CMOS/CCD); computer; fluorescent beads in gel	[95, 97, 100–102]
µFSA (microforce sensor array)	Displacement of pillars	Fluorescent pillars; microscope; CMOS/CCD; computer	[103, 104]
AFM (atomic force microscope)	Bending of cantilever	Cantilever; microscope; CMOS/CCD; workstation; Piezo actuator	[105, 106]
OT (optical tweezers)	Displacement of trapped bead	Laser; microscope; CMOS/CCD; workstation; Piezo actuator	[107–109]
MT (magnetic tweezer)	Displacement or torque of bead	Magnets; microscope; CMOS/CCD; Piezo actuator; workstation	[110, 111]
MPA (micropipette aspiration)	Change of suction pressure	Micropipette; microscope; computer; CMOS/CCD	[112–115]
MPS (micropipette force sensor)	Deflection of micropipette	Micropipette; microscope; CMOS/CCD; computer	[116–119]
Flow chamber and microfluidic chip	Change of ρ, V, µ, L	Flow chamber; pump; microscope; CMOS/CCD; computer	[120–125]
RT-DC (real-time deformability cytometry)	Deformation, cell size	Inverted microscope; high-speed CMOS camera; LED lamp	[126, 127]
Super-resolution microscope	Change of fluorescence intensity	Confocal microscope; CCD camera system; Image sensors	[128, 129]
Biosensors (i.e. TGT, hairpin TS)	Change of fluorescence intensity	Nanosensor; microscope; CMOS/CCD; computer	[130–132]

Cells exert very small forces in the range of pN to nanonewtons (nN). Therefore, to monitor the forces involved in specific cellular processes, it is essential to use well-designed, sufficiently sensitive tools tailored to research goals. Force sensing in cells may be mediated by membrane receptors, prompting researchers to select ligands such as the tripeptide RGD for integrins αvβ3, α5β1 and αIIbβ3 [91]. Alternatively, relevant cells can be studied as a whole unit at the single-cell level, allowing for the recording of changes in shape, volume and other mechanical parameters [92, 93]. The following sections will outline the analytical approaches successfully applied in measuring biological forces, with key features summarized in Table 2.

**Table 2.** The comparison among various devices for cell mechanical study

	Spatial resolution (nm)	Force resolution (pN)	Detecting range (pN)	Modification requirement	Application in cell mechanics	Limitations or disadvantages	References
TFM (traction force microscope)	400–7000	≈10^3^	10^3^–10^5^	Fluorescent beads embedment	Cell traction in 2D/3D matrix; single and multi-cells;	Complexity of data interpretation; resolution limitations	[133–136]
µFSA (microforce sensor array)	<1	≈10	50–10^5^	Pillar modified with fluorescent Fibronectin	Cell traction in 2D/3D; single and multi-cells; basal force	Limited sensitivity and resolution; complex calibration	[104, 133, 135]
AFM (atomic force microscope)	0.5–1	≈1	10–10^4^	Cantilever modified with ligands	Cell stiffness; ligand–receptor interaction; single cell; basal force	Limited scan area; slow scanning speed	[106, 135, 137–142]
OT (optical tweezers)	0.1–2	<1	0.1–10^2^	Beads modified with ligands	Cell rheology; ligand–receptor interaction; single cell;	Limited size and shape of target; photodamage and heating; speed limitations	[133, 135, 137, 143]
MT (magnetic tweezer)	1–10	<1	0.001–10^2^	Beads modified with ligands	Bond’s torque; ligand–receptor interaction; single-cell mechanics	Limited force range; limited spatial resolution	[133, 135, 137]
MPA (micropipette aspiration)	25	NA	0.3 (0.1–10 mbar)	Beads modified with ligand	Ligand–receptor interaction; single-cell mechanics	Low throughput; limited precision in force measurement; limited spatial resolution	[144–147]
MFS (micropipette force sensor)	1	1	1–10^5^	–	Cell adhesion force; cell membrane tension; micromechanics of the metaphase spindle, single actin filament, and single kinesin	Low throughput; limited precision in force measurement; limited spatial resolution	[116–119, 148, 149]
Flow chamber or microfluid chip	–	–	–	–	Cell adhesion; lipids/macromolecules distribution in vessels; cell response to fluid flux; cell mechanical heterogeneity	Used in biochemically study cell response to shear stress	[124, 150–151]
RT-DC (real-time deformability cytometry)	340	≈10^6^	–	–	Cell rheology, cell deformation	Limited to suspension cells; limited sensitivity to soft cells; limited detection of mechanical heterogeneity	[126, 127]
Super-resolution microscope	–	–	–	Fluorescent protein/fluorophore-conjugated protein	Cytoskeletal protein dynamics	Photobleaching and phototoxicity; limited quantification of mechanical properties	[128, 129]

### Traction force microscope

Traction force microscopy (TFM) was originally conceived using elastic silicon rubber as the cell adhesion substrate. As the cell wrinkles and folds, the rubber deforms and makes the force ‘visible’ [94].

**Figure 4.**
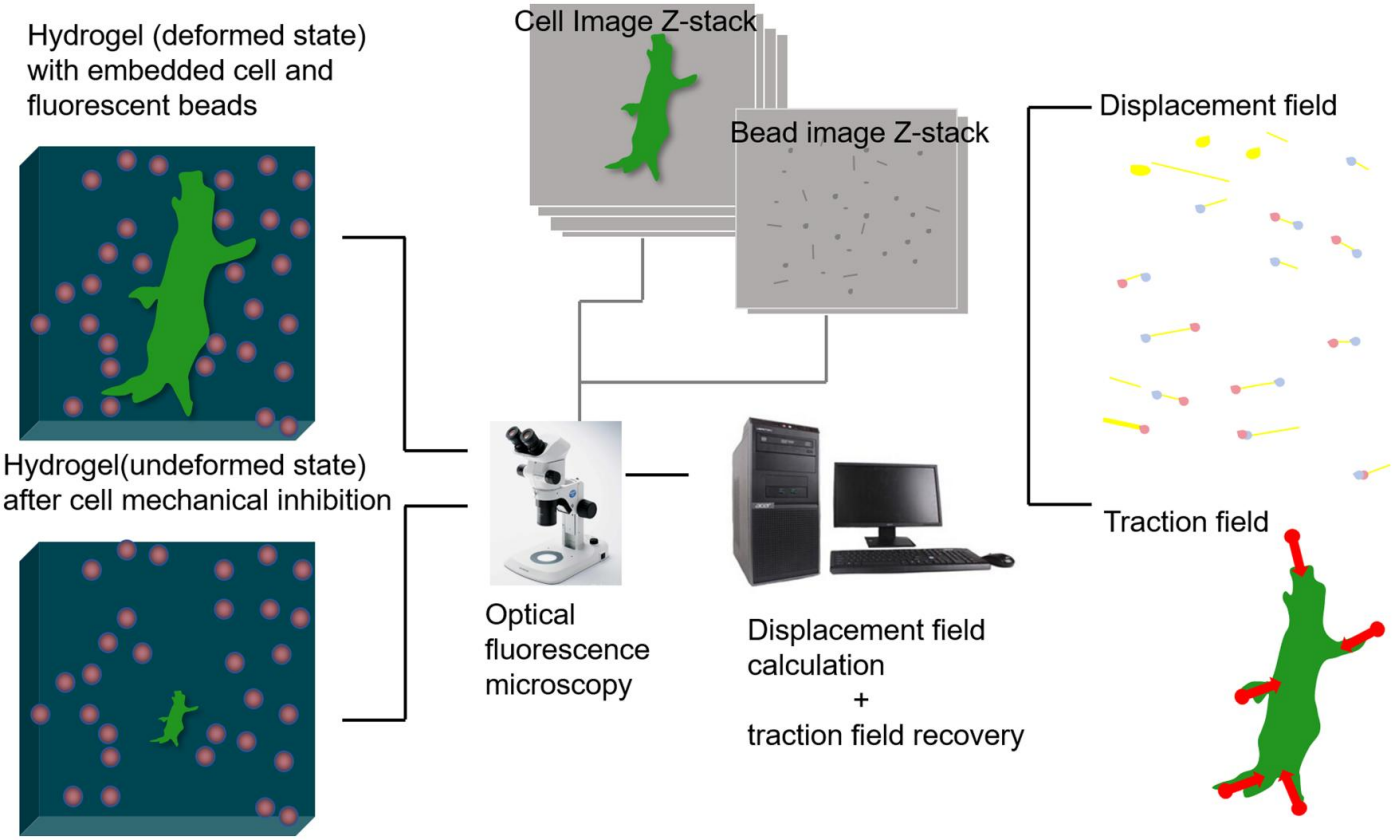
The principle of TFM. The cell displacement and bead dislocation were imaged in Z-stack mode [154]. The raw images were used to interpret the force at certain region, where the stress-strain relationship could be calculated according Hooke’s law. We acknowledge permission from the copyright clearance center of the RSC to reuse the article [154].

In current devices, cells are plated onto or embedded in polyacrylamide (PAA) gels containing fluorescent beads. The positions of these beads can be monitored using a fluorescence microscope (Figure 4). To calculate the forces generated by individual cells, two methods are commonly used. The recently developed approach involves directly recording the displacement of the beads, which requires high-resolution and high-accuracy measurements of the displacement field. The strain field (***ε***) is then calculated from the measured displacement field (***u***) using a linearized expression that applies to small strains:

$$\varepsilon_{i,j}=\frac{1}{2}\left(\frac{\partial u_{i}}{\partial x_{j}}+\frac{\partial u_{j}}{\partial x_{i}}\right),95$$

with *u* = (*u_1_, u_2_, u_3_*) and *x* = (*x_1_, x_2_, x_3_*).

The stress field σ can thus be obtained using Hooke’s law, which governs linear elasticity through a constitutive relationship.

σ=cε

where *c* is the stiffness tensor, which describes the substrate properties.

The second method of calculation employs a convolution approach to characterize the displacement field *u* and illustrate cell tractions *t*, as demonstrated by the Green’s function presented below:

u(r)=∫dr′G(r−r′) t(r′).[96]

G explains the impulse response, which is the output of a linear system under zero initial conditions and a unit impulse function as input, to a point load. Equation 3's integral can be seen as a summation, where each displacement *u* at position *r* = (*x, y, z*) reflects the cumulative impact of all traction forces *t* operating at *r*' = (*x', y', z'*).

Furthermore, Butler *et al*. [97] pioneered the solution of the Fredholm integral for TFM in Fourier space, introducing the Fourier transform traction cytometry (FTTC) method. This method remains the predominant approach for force reconstruction, offering computational efficiency. Nonetheless, FTTC is susceptible to experimental noise in the displacement field, potentially leading to deviations in the reconstructed tractions. Accurate estimation of traction force can be realized by various and complementary methods such as the finite element method, which allows researchers to monitor cell forces in a 3D matrix by embedding cells into elastic polyethylene glycol (PEG) gels.

Whole-cell force mapping using TFM typically has a relatively low spatial resolution. To address this, transparent PAA gels were introduced alongside genetically modified cells that can be observed under a fluorescence microscope. This enhancement allows for improved resolution, enabling the analysis of cell focal adhesions and the cytoskeleton. The stiffness of PAA gels can be adjusted from 1.2 to 100 kPa to mimic the elastic properties of surrounding tissues found in nature [98]. To further enhance spatial resolution and accuracy, multiple fluorophores (beads) at high densities are often dispersed onto the substrate. Cross-correlation calculations can then be applied to increase precision in measuring bead displacements in each channel [99]. Microcontact printing techniques have also been adapted in order to prepare regular arrays of embedded fiducial markers with a homogenous distribution. However, constructing matrices and calculating relevant equations typically require extensive computational resources.

Recent advancements aim to improve traction force analysis in two key ways: first, by integrating super-resolution microscopy with TFM, enabling the detection of displacements at nanometer scales; and second, by updating reconstruction algorithms to enhance sensitivity and accuracy. The fluctuation-based super-resolution algorithm has recently increased both trackable bead density and tracking accuracy in TFM experiments [155]. This technique has enabled the examination of filopodia alignment—tiny protrusive structures in cells—advancing our understanding of cell force sensing. Additionally, 2.5D astigmatic TFM measures both lateral and axial components of displacement. By combining TFM with fast 2.5D astigmatic imaging and total internal reflection fluorescence structured illumination microscopy (SIM), this approach achieves a tenfold increase in temporal resolution compared to super-resolution methods for 3D mechanical force quantification [156].

TFM is particularly useful for studying cell migration, contraction, invasion and focal adhesion organization. For example, real-time TFM has been used to analyze epithelial bladder cancer cells with varying degrees of invasiveness [157]. The invasive bladder cancer cell line T24 requires less traction force to migrate compared to the less invasive RT112, which is attributed to a large actin rim at the cell edge of RT112, while T24 exhibits a more organized cytoskeleton. Also, TFM has been employed to track the maturation of iPSC-derived cardiomyocytes at varying calcium concentrations [158]. Human-induced pluripotent stem cells cultured for 1–3 months demonstrated greater contraction forces compared to those cultured for 3 weeks, highlighting the importance of physiological calcium concentrations for optimal contractility. Using microparticle TFM, Theriot *et al*. investigated phagocytic engulfment and force dynamics in the T-cell immunological synapse [159]. When combined with confocal microscopy, this method enabled extraction of information from particle boundaries, achieving super-resolution accuracy with local deformations measured to within <50 nm. Lee *et al*. introduced refractive-index TFM (RI-TFM), a novel technique that enables simultaneous quantification of cellular volumetric morphology and traction forces with a temporal resolution of 0.5 Hz using a high-speed illumination scheme [160]. Enhanced by a constrained total variation-based deconvolution algorithm, RI-TFM provides a sensitivity of 0.55 Pa for shear traction and 1.59 Pa for normal traction on a 1-kPa hydrogel substrate. Leveraging this approach, we analyzed the dynamic spatiotemporal distribution of proteins and traction forces in three distinct cell types: NIH-3T3 fibroblasts, MDCK epithelial cells and CD8^+^ T cells. These techniques show great promise for studying inter- and intracellular forces.

### Microforce sensing arrays

**Figure 5.**
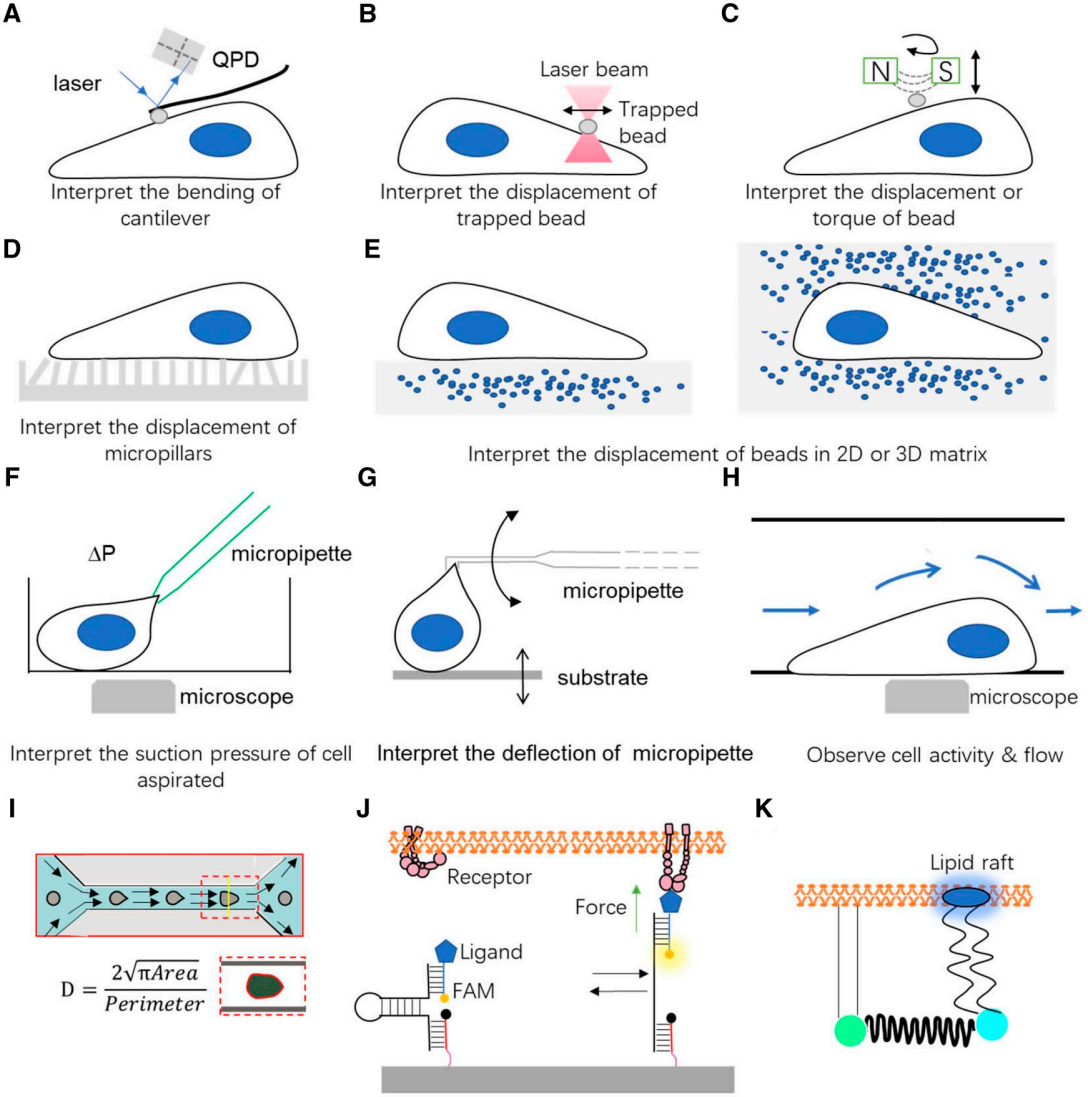
Schematic of different analytical methods for cell force studies. (**A**) Atomic force microscope probing the cell stiffness [106]. (**B**) Optical tweezers measuring the dynamics of the cell–ligand interactions via trapped beads [143]. (**C**) Magnetic tweezer records the torque and normal tension between beads and ligands [110]. (**D**) µFSA monitors the displacement of micropillars as they are pulled by the cell [143]. (**E**) TFM detects the cell force in 2D and 3D, depending on the displacement of labeled beads [103, 104]. (**F**) Micropipette aspiration can quantify the cell–cell interaction, the experienced force will be computed according the suction pressure [114]. (**G**) Micropipette force sensor enables the measurement of microalgae adhesion force onto substrate [118]. (**H**) Fluidic systems favor the observation of cell status in the flow chamber of various dimensions [122]. (**I**) Real-time deformability cytometer [126]. (**J**) Force tension sensors based on DNA fragments [161]. (**K**) Membrane tension sensor based on elastic peptide [162].

TFM typically requires substantial computing resources. An alternative method, known as microforce sensing arrays (µFSA) (Figure 5D), uses the elastic modulus of the substrate. In this approach, a well-patterned micropillar system is created through microfabrication techniques and labeled with fluorescent ligands such as fibronectin. The deflection of the pillars reflects the forces applied by cells seeded on top. The following equation [103] provides a reference for calculating the forces: a deflection on a cylinder of radius r and length L corresponds to a force *F*:

$$F=k\cdot x=\left[\left(3\pi Er^{4}\right)/\left(4L^{3}\right)\right]x$$

where *E*, *k* and *x* are, respectively, the *Young’s* modulus, the spring constant and the deflection of the pillar.

The fabrication of micropillar arrays typically involves using negative molds. Photolithography is an effective method for creating ‘pits’ on silicon wafers, which serve as these molds. After salinization, a layer of polydimethylsiloxane (PDMS) is applied to the mold via centrifugation and then cured. The final step involves functionalizing the micropillars with cell ligands or fluorescent labels, which can be achieved through microcontact printing using PDMS molds and liquid drops. Alternatively, arrays can be prepared directly on silicon wafers by applying a uniform photoresist coating, followed by exposure to ultraviolet (UV) light through a photomask [163]. The photomask defines the size and distribution of the pits. After development and plasma etching, the desired pits are formed. A chromium mask can be used to produce micropillars with a higher aspect ratio due to its greater resistance to plasma etching. Before measuring cell traction forces, the mechanical properties of the micropillars must be characterized. Dimensions can be measured using scanning electron microscopy or AFM. The Young’s modulus of the materials can be quantified through bulk measurements [164]. Also, AFM in contact mode makes it possible to directly measure the stiffness of the micropillars [165].

µFSA has been applied in cell mechanical studies for a long period and interesting findings elucidate the processes of cell mechanobiology. Ladoux *et al*. have used the µFSA system to decipher the relationship between focal adhesion (FA) architecture, molecular forces within FAs and cell traction forces [166]. They pinpointed that molecular tension within individual FAs follows a biphasic distribution resulting from the proximal (toward the cell nucleus) and distal (toward the cell edge) ends and proposed a model regarding the FA dynamics and tension changes therein. µFSA may give a reliable force measurement on the order of ∼1 nN. Bashour *et al*. delineated the contribution of T-cell receptor (TCR) and CD28 during T-cell activation by µFSA [169]. Liang *et al*. measured the average force per platelet to be 2.1 ± 0.1 nN with this design [170]. Based on micropillar or nanopillar substrates, Doss *et al*. investigated the interplay between matrix rigidity and the rheological properties of the cytoskeleton, where they demonstrate cell shape changes and polarity is modulated through cell-scale mechanosensing and local rigidity sensing at FAs [171]. In addition, the topographical effect of micropillars-based arrays on cells has been studied well. Light-responsive nanostructures created by an azopolymer were reshaped from vertical pillars to elongated vertical ones upon green light illumination. The increased membrane curvatures at bar ends lead to local accumulation of actin fibers and Arp2/3 complex [172]. Hansel *et al*. found that nanoneedles made of silicon interfere with cell mechanical translation, and their results elucidated that these nanoneedle arrays inhibit focal adhesion maturation, decrease cytoskeletal tension, and lead to remodeling of the nuclear envelope at sites of impingement [173]. Similarly, Bucaro *et al*. showed that the shape and alignment of mesenchymal progenitor stem cells (C3H10T1/2) can be precisely controlled by adjusting the spacing and geometry of nanopillars (NPs). They identified a characteristic spacing (∼2 μm) using high-aspect-ratio, geometrically soft NPs, which promoted pronounced polarization and the growth of axon-like extensions aligned with the NP lattice [174]. Furthermore, Hanson *et al*. found that the spacing of NP arrays had a more pronounced impact on nuclear deformation compared to the pillar radius, while the height of the nanopillars exerted a moderate effect on nuclear deformation [175].

### Optical tweezers

Optical tweezers (OTs) were first designed by Arthur Ashkin and colleagues in 1970 [176], which is suitable for monitoring the forces between beads functionalized with ligands and cell membrane receptors, as well as for assessing the microrheological properties within cells. A typical OT setup uses a laser beam to create a light field with a gradient intensity capable of trapping dielectric particles or beads (such as silicon or polystyrene) along the laser path. The pulling force positions the dielectric particle toward the focal point, while the scattering effect generates a pushing force that drives the particle away from the focus. Achieving this balance requires a tight focus, with a significant portion of the incident light entering at large angles [177].

The effective traps must account for the refraction index, particle geometry and the gradient of the laser beam [178]. The dielectric particles act as handles that can be modified with ligands to interact with specific receptors on cell membranes or within cells. The forces exerted by cells to displace the trapped beads can be quantified by monitoring bead movement (Figure 5B). Calibration is a crucial step before measuring mechanical stimuli on cells. The thermal fluctuation method is commonly used to calculate both trap stiffness and the conversion factor for the position detector. The instrument pre-loads the force-displacement algorithm, allowing for easy observation of bead movement using a quadrant photodiode displayed on a computer monitor.

**Figure 6.**
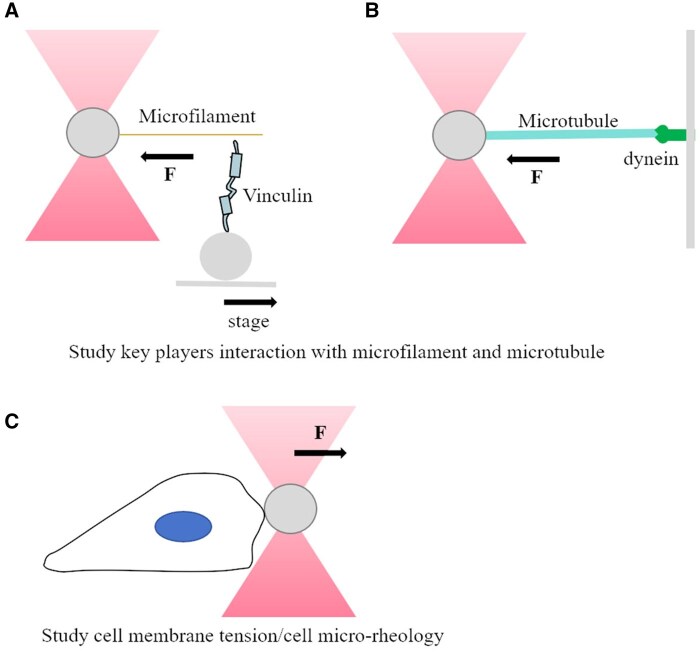
Optical tweezers in cell force sensing. (**A**) A single-molecule optical trap assay is used to investigate the bonding property between vinculin and actin filament [49]. Beads are immobilized with T12 vinculin and fixed on a stage. The stage is translated which leads to displacement of trapped beads. The authors suggest that the directional and force-stabilized binding of vinculin to F-actin may help understand how adhesion complexes keep front-rear asymmetry in migrating cells. (**B**) Studying microtubules dynamics and interaction with dynein. Microtubules nucleate from an axoneme, linked to a bead and held by a ‘keyhole’ optical trap, grow against a microfabricated barrier. Microtubule-associated proteins (MAPs) and motor proteins may be added in solution and a mechanical signal is generated [167]. (**C**) Studying cell membrane tension/cell microrheology. Huh7 cell membrane tension is measured by tether-pulling. Initially, the bead is trapped by; after contacting with cell membrane for 30 seconds to form a lipid tether, the bead is pulled away from the cell [168].

This technique enables the measurement of cell mechanics with high temporal and spatial resolution, achieving piconewton forces and sub-nanometer precision (see examples in Figure 6). The temporal resolution is on the microsecond scale. Unlike AFM and magnetic tweezers (MTs), OTs can also measure intracellular interactions, such as myosin movement along microtubules. However, concerns about photodamage to cells due to local heating from the laser may affect observations [179]. Strategies to mitigate photodamage have been explored, including the use of near-infrared lasers, two divergent laser beams instead of a single focused beam, and even heavy water as the medium [180]. Additionally, Peterman *et al*. examined the enzymatic activities and associated changes in the local viscosity of the microenvironment.

Bustamante *et al*. reviewed various setups of OTs, including standard OTs, dual-trap OTs and angular OTs, all of which enhance our understanding of biomolecular forces at the single-molecule level. By a single-molecule optical trap assay, Huang *et al*. proved vinculin–F-actin forms a directional catch bond [49]. They tested forces ranging from 1 to 30 pN and found that the lifetime of the vinculin–actin filament bond varied with the direction of stretching. Bonds formed at the pointed (−) end of the actin filament exhibited a ∼10-fold longer mean lifetime compared to those at the barbed (+) end, indicating an asymmetric binding property dependent on directionality. Also, cell elasticity and microrheology measurements have been calibrated by OT under various conditions [143, 181, 182]. In immune cells, Kim *et al*. investigated the interaction between major histocompatibility complex molecules (pMHC) and TCR, finding that oscillating forces of 50 pN could trigger Ca^2+^ influx in T-cell Ca^2+^ influx [183]. It was later shown that shear forces of approximately 10 pN could activate T lymphocytes through TCR–pMHC interactions [184]. OTs have been integrated with other setups, such as fluorescence microscopy and microfluidic systems, to collect more details during cell responses under various conditions. For single-cell microrheology measurements, a feedback-tracking approach has been devised to monitor particle probes inside fibroblasts and HeLa cells, and then the data analysis by fluctuation-dissipation theorem within positional feedback reveals the glassy dynamics of the cytosol [185]. OT has been applied to study the mechanics of cellular organelles including nucleus, mitochondria, the Golgi apparatus and the ER [186–188]. Recent advances in OT-based active microrheology have even enabled the identification of different cell types based on their mechanical states [189]. With their high spatial and temporal sensitivity, OTs serve as powerful tools for decoding the complexities of mechanobiology and cell signaling.

### Magnetic tweezer

**Figure 7.**
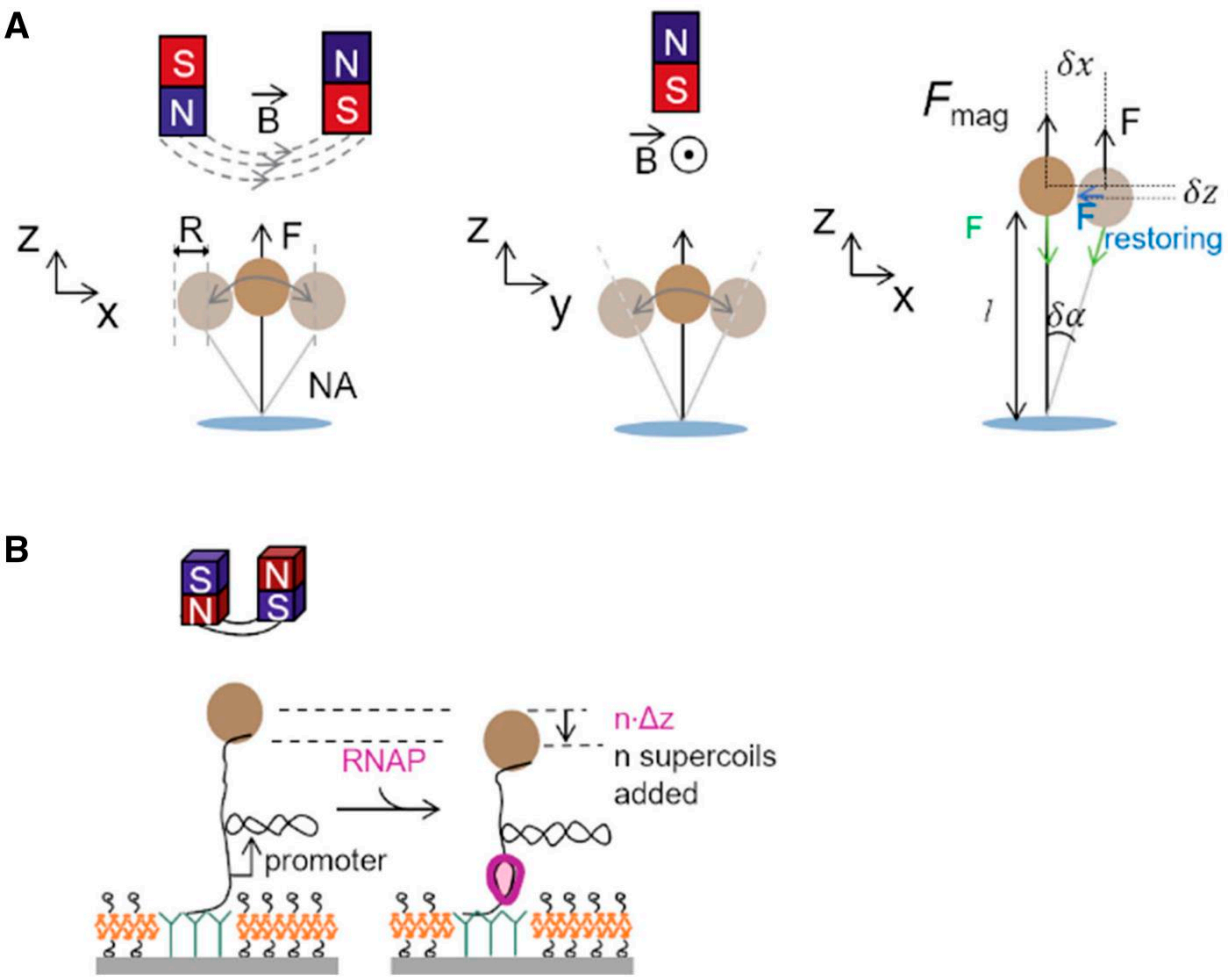
The principle of MT. (**A**) The magnetic beads movement in a magnetic field. The displacement can be divided in to δx, δy, δz. (**B**) Measuring the DNA plectonems formation upon positive supercoiling using magnetic tweezer. RNA polymerase (RNAP)–promoter open complex formation on a positively supercoiled DNA. Promoter opens DNA and n positive supercoils addition occur, and the bead downward movement was recorded by n·Δz [195].

MTs apply mechanical stimuli to a cell or a tethered peptide with a very high force and temporal resolution (Figure 5C). In the setup, ferrite nanoparticles are magnetized and controlled by an external applied magnetic field, the force is a function of the distance between the nanoparticles and electromagnets under paired magnetic field (Figure 7). The force experienced by a magnetic particle in a magnetic field is the magnetic force, $\vec{m_{\mathrm{sat}}}$ is the saturated magnetization of the particle, and $\vec{B}$ is the magnetic field.

In the MT experiment, the force can be calculated from the magnitude of the horizontal bead fluctuations, *<δy^2^>*. According to the Equipartition Theorem, *k_y_<δy^2^> = kT*, where *k_y_* is the horizontal stiffness of the trap, *T* is the temperature and *k* is the Boltzmann constant. For a bead tethered to the surface via a single linkage, the stiffness is proportional to the applied force, *k_y_* = *F/l*, where *l* is the length of the tether, *r* its radius, leading to the following relationship: $F_{\mathrm{horizontal}}=\frac{\mathrm{kTl}}{<\delta y^{2}>}$

Also, the vertical magnetic force *F*_vertical_ may then be evaluated through the simple formula, $F_{\mathrm{vertical}}=\frac{\mathrm{kTl}}{<\delta x^{2}>}$ where $<\delta x^{2}>=\frac{kT(l+r)}{F_{\mathrm{mag}}},\;<\delta y^{2}>=\frac{\mathrm{kTl}}{F_{\mathrm{mag}}}$ [190]

Thus, the force can be readily computed once the length of the tether and the variance of fluctuations are known. The imaging/tracking system is used to monitor the position of a micrometric magnetic bead immersed in an aqueous solution. The displacement could be obtained for force calibration. Since the magnetic beads integrate and respond directly to the magnetic forces, it arms the MT with the ability to displace along multiple axes of movement. Thus, one can investigate various types of forces including rotation and multiple directions of translation [191].

As for the magnetic field, it can be categorized into static and dynamic types. Herein the dynamic magnetic field is often generated by displacing permanent magnets or electromagnets and applied in MT configuration [192]. To produce a strong magnetic gradient, electromagnets with sharp tips are employed, and their magnetic field distribution can be quantified, showing a gradual reduction in intensity with increased distance from the tip. Typically, pairs of electromagnets are used to create a parallel and consistently reduced magnetic field over a relatively wide area. Magnetic beads or particles directly integrate and respond to these magnetic forces, enabling MTs to displace along multiple axes. This allows for the investigation of various types of forces, including rotation and translation in multiple directions [191]. To respond effectively to the electromagnetic field, the materials used for the beads or cubes must be carefully selected. The shape of the particles also influences their magnetic properties; for instance, cubic particles have disordered spin states due to their sharp corners, while spherical beads have evenly distributed spins [193]. Ferrite compounds are commonly chosen for magnetic nanoparticles because they respond predictably to external magnetic fields, allowing for the creation of a locally controllable magnetic field.

The MT has been utilized to study cell mechanics, focusing on protein–protein interactions *in vitro* and ligand–receptor interactions *in vivo*. *In vitro*, Le *et al*. demonstrated the high mechanical stability between vinculin and its binding partners (talin and α-catenin) using MT, finding lifetimes exceeding 1000 seconds at forces up to 10 pN, and lifetimes ranging from seconds to tens of seconds at 15–25 pN. This supports an efficient force transduction model along vinculin–talin/α-catenin in cell–matrix and cell–cell adhesions [194]. Additionally, Tapia-Rojo *et al.* uncovered remarkable complexity in the equilibrium folding and unfolding energy landscape of the talin R3^IVVI^ mechanosensory domain. Their findings indicate that, in the most frequent folding scenario, the four helices of the talin R3^IVVI^ domain fold cooperatively within the experimental time resolution (∼1–2 ms at a sampling rate of ∼1500 Hz), yielding an effective two-state energy landscape. Considering that talin requires ∼700–1500 seconds to dissociate from FAs [196] and that focal adhesion maturation can span several minutes [197], it is plausible that the low-probability states identified in talin R3 are transiently populated during the processes of focal adhesion assembly and maturation. These rare states, where stretched talin molecules are unable to bind vinculin or effectively interact with the actin cytoskeleton [15], may underlie the broad timescales observed in cellular mechanotransduction [198].

*In vivo*, the genetically modified magnetically sensitive actuator known as ‘Magneto’, which combines the cation channel TRPV4 with the paramagnetic protein ferritin, was shown to induce calcium influx under magnetic forces, as evidenced by calcium imaging [199]. This work highlighted the role of striatal dopamine receptor 1 neurons in mediating reward behavior in mice and contributed to methods for remotely controlling circuits in studies of animal behavior. Additionally, combining magnetic particles with hydrogels allowed for the quantification of the relationship between stiffness and forces in tumor colonies and embryos [200]. Microfluidic technologies facilitated the assembly of microgels, such as an RGD-conjugated stiff PEG round microgel embedded with a magnetic microrobot, which detailed cell traction forces and stiffness in 3D. The microrobot acted as both a force sensor and a stiffness probe, revealing that malignant tumor-repopulating cell colonies altered their modulus without changing traction forces in response to varying 3D matrix elasticities. Furthermore, small magnetic particles functionalized and injected into cells enabled the investigation of intracellular mechanical properties, including cytoplasmic viscosity, vesicle transport, nuclear rheology and mitosis [39, 201–203]. Garzon-Coral *et al*. measured the spindle centering force using MT and demonstrated that the centering spring stiffness (16 pN/mm) ensures the spindle positioning precisely against thermal and other ambient fluctuations, and critically guarantees asymmetric cell division [203]. Also, Collins *et al*. elicited the force transduction on platelet EC adhesion molecule-1 via integrin-Rho pathway by MT [204]. Human breast carcinoma cells turned softer upon exposition to higher temperatures which was also demonstrated by MT measurement [205]. The growing discoveries by manipulating magnetic-based instruments have been recognized as promising platforms to unveil the mechanotransduction of relevant biosystems.

### Atomic force microscope

**Figure 8.**
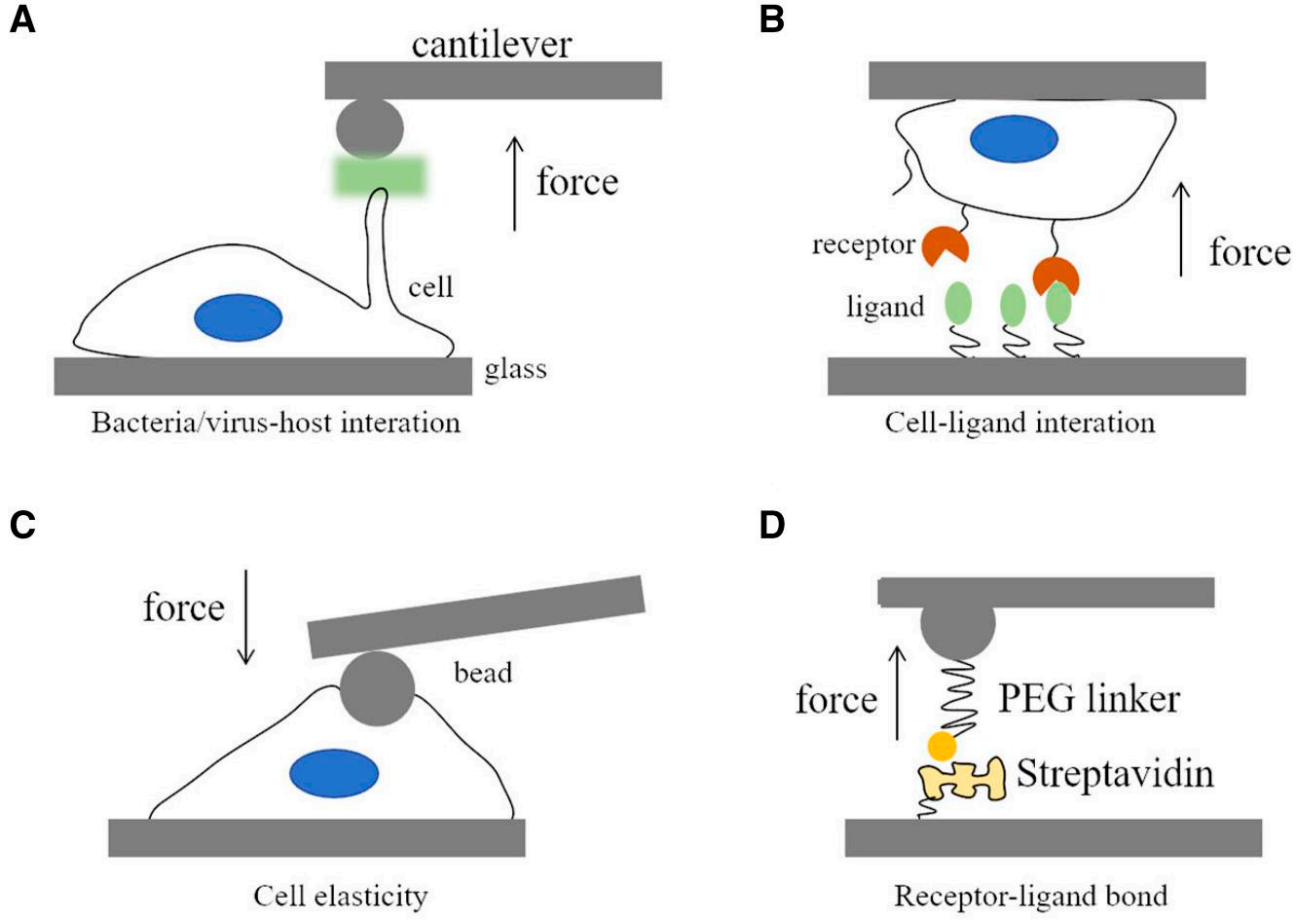
AFM used in cell force sensing. (**A**) Bacteria–host binding process investigation where WT LGG bacterium is attached to the cantilever probe and brought into contact with cell surface. The formation of membrane nanotethers and bonding property are investigated [207]. For virus–cell binding process [208], this beginning step of binding between the virus to the cell involves EnvA glycoproteins to complementary tumor virus A (TVA) receptors. The AFM cantilever conjugated with virus approaches and retracts from the cell. (**B**) Cell–ligand interaction where a single fibroblast was immobilized on ConA- or VN-coated cantilever, approached to the substrate-coated support, and retracted vertically after a few seconds contact time. The adhesion force between fibroblast and support was measured during this process [52]. (**C**) Cell elasticity measurement where the cell is indented by a bead probe, and the mechanical response of contacted areas are recorded. Cortical stiffness of a cell is analyzed according to force–distance curves generated in this assay [151]. (**D**) Ligand–receptor interaction *in vitro*. Biotin is attached to the cantilever and streptavidin to the support. The force curve monitors the separating process along the chain, which includes the initial elongation of PEG linker as well as eventual rupture force of the bond [209].

AFM represents a high-resolution form of scanning probe microscopy, employing a cantilever with a sharp or colloidal tip to traverse the sample surface [206] (Figure 8). During scanning, various forces, such as Van der Waals forces, electrostatic forces and hydrophobic/hydrophilic interactions, induce deflections in the cantilever. These deflections are detected by a laser reflected onto photodiodes, producing an output signal proportional to the cantilever's bending. This signal is processed to determine the vertical displacement of the cantilever and transmitted to a scanner, which adjusts the probe's height across the surface. By regulating the scanner’s height, a 3D topographical map of the sample is generated. In force measurement mode, the deflection of the cantilever (*Z*_c_) is plotted against the Piezo position (*Z*_p_), which is perpendicular to the surface. Converting *Z*_c_ and *Z*_p_ into force and distance values yields a force-versus-distance curve. The force, *F*, is calculated by a position-sensitive detector. When force bends the cantilever, the reflected light beam shifts by an angle twice the change in the end slope, d*Z*_c_/d*X*. For a cantilever with a rectangular cross-section (width *w*, length *L* and thickness *a*), the change in the end slope is expressed as

$$\frac{dZ_{c}}{dX}=\frac{6FL^{2}}{Ewa^{3}}[210]$$

where *E* denotes Young’s modulus of the cantilever material and *F* represents the force applied to the cantilever’s end in the normal direction. The signal detected via the optical lever technique is directly proportional to the end slope of the cantilever, hence providing the cantilever’s deflection as below:

$$Z_{c}=\frac{4FL^{3}}{Ewa^{3}}=\frac{2}{3}L\frac{dZ_{c}}{dX}[210]$$

In typical experiments, physiological conditions are simulated by keeping samples in appropriate buffers. An environmental control system can be used when needed to maintain the pH, temperature, atmosphere, humidity, and energy requirements. For example, in cell indentation tests, force–distance (FD) curves are generated, and cell elasticity is then analyzed using models such as the Hertz model [143], which assumes that samples are purely elastic, infinitely expandable and lack substructures. To enhance data accuracy, researchers may also apply the Johnson–Kendall–Roberts model or the Derjaguin–Muller–Toporov model, which are suitable for analyzing interactions between spherical probes and flat surfaces [211, 212]. A constant force or indent depth mode in cell indentation test facilitates the analysis of the dynamics of the cantilever–sample interactions. The stiffness or spring constant of cantilevers is fundamental to investigating interactions between the cell surface and cantilever tip. Note the cantilever with spring constant similar to those of the samples in biology is a critical factor as it gives an observable deflection in sampling [143]. Also, at least 400 nm indented depth is highly recommended in nanoindentation test [213]. Additionally, the loading rate is critical during calibration and measurement; thus, force comparisons are meaningful only at consistent loading rates. Consequently, ligand–receptor interactions are often assessed across varying loading rates or pulling speeds to examine their dynamic mechanical properties.

AFM enables the studies of mechanics at the cellular as well as the single-molecule level (see examples in Figure 5). So far, this technique has already enriched our knowledge of cell stiffness, cell rheology and cell mechanosensation [214]. AFM-based nanoindentation methods allow for localized force application in small contact areas. *In vivo,* AFM has been used to study the mechanical effects on neuronal growth by locally perturbing brain tissue stiffness, revealing that alterations in brain stiffness lead to axonal pathfinding errors. This suggests that growing neurons respond to mechanical signals in addition to chemical cues [215]. Using AFM, researchers have also calibrated the stress stages as a cell membrane is pierced by a nanotip [123] and examined the frequency-dependent viscoelastic behavior of NIH 3T3 fibroblasts [216]. The apparent stiffness of mammalian cells, as measured by AFM, typically ranges from 1 to 100 kPa [151, 217]. Combining AFM with traditional microscopy enables simultaneous observation of applied forces and cell responses [208, 218, 219]. The Sivasankar group has focused on the mechanical interactions between E-cadherins for years, finding that E-cad bonding properties may play a role in cancer cell migration [209, 220]. The Müller group has explored cell–ligand and cell–virus interactions from a physical perspective [52, 221]. Recently, native ECM fragments cut by laser microdissection have been attached to AFM cantilevers and brought into contact with cells, demonstrating the feasibility of investigating cell–microenvironment interactions and early mechanotransductive processes using force spectroscopy [222]. Another significant advancement is the standardized nanomechanical AFM procedure method, which eliminates errors in determining deflection sensitivity and cantilever spring constants. It does this by accurately calculating deflection sensitivity based on thermal fluctuations of the free cantilever, using an independently determined spring constant value [223]. To investigate the role of mechanical cues in axon growth, Koser *et al*. employed *in vivo* AFM (iAFM) alongside biological manipulations, including Piezo1 knockdown and pharmacological blocking of mechanotransduction [215]. AFM has also been adapted to actively confine cells at a cellular scale, enabling studies of cell migration and movement [224]. Furthermore, confocal video microscopy combined with AFM-based force spectroscopy has been used to monitor contractile forces and myosin cytoskeleton dynamics [225]. High-throughput AFM approaches now allow simultaneous analysis of the morphological and nanomechanical properties of hundreds of extracellular vesicles within hours [226].

### Micropipette aspiration

**Figure 9.**
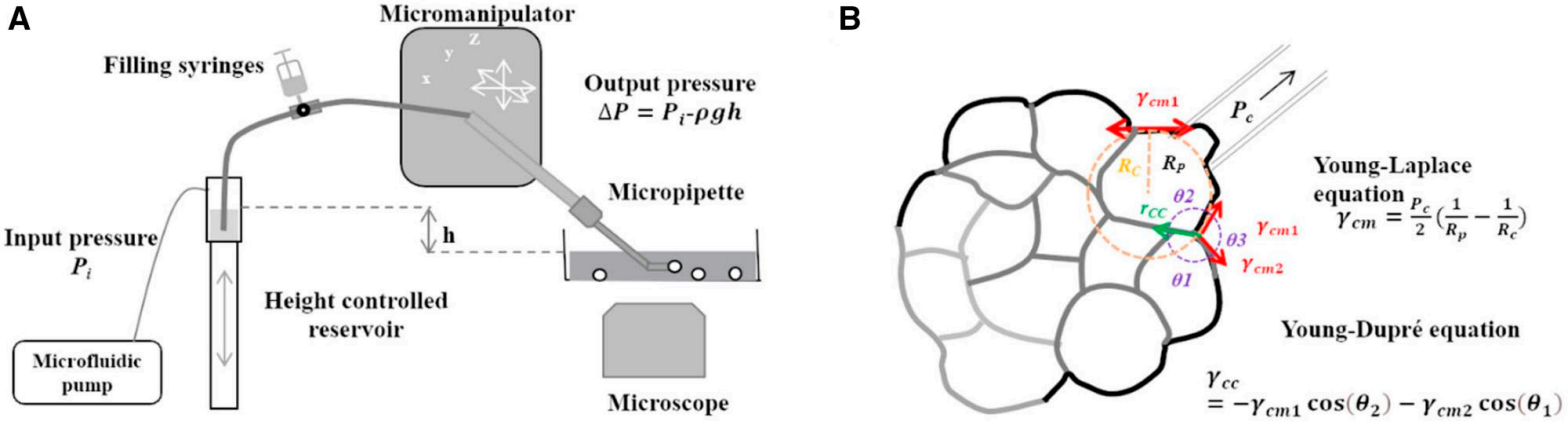
The micropipette aspiration setup and cell membrane tension measurement by MPA. (**A**) Schematic diagram. The micropipette is connected to a liquid reservoir, mounted on a stage and the pressure can be modulated precisely. (**B**) The equation used in cell membrane tension analysis. The parameters and equations are explained in the text. (We acknowledge permission from the Copyright Clearance Center of the Elsevier to reuse the article [228].) The micropipette approach in MPA demonstrates its application in cell mechanics studies, but the force-sensing method of micropipette force sensing (MFS) is conceptually different. Instead of probing suction pressure, MFS measures the deflection of a micropipette force sensor to assess force using Hooke’s law [119, 243] (Figure 5G). This technique evolved from earlier flexible needles used for force sensing in biological cells and molecules [118, 149, 244, 245].


Developed in 1973, micropipette aspiration (MPA) has evolved over decades, significantly enhancing its effectiveness for studying cell mechanics [227]. MPA involves holding the cell membrane with a micropipette using suction (Figure 5F). The micromanipulator adjusts the suction pressure, while an imaging system captures cell shape changes in response to the applied force (Figure 9). Suction pressure is calculated based on the height difference between the micropipette tip and the top of the reservoir, and it can be finely adjusted within a range of 1 Pa to several kPa [145]. The microscopy system monitors cellular changes at the micropipette tip, providing data on cell volume changes and pressure variations. The surface tension at the cell–medium interface $\gamma_{\mathrm{cm}}$ of the cell is calculated using the Young-Laplace equation:

$$\gamma_{\mathrm{cm}}=\frac{P_{c}}{2}(\frac{1}{R_{p}}-\frac{1}{R_{c}}),$$

where *R*_c_ represents the resting radius of curvature of the interface in contact with the micropipette, while *P*_c_ denotes the critical pressure at which the cell deformation extends to *R*_p_, the micropipette radius.

The tensions of interfaces $\gamma_{\mathrm{cc}}$ for which the tensions of two contacting interfaces are known, is calculated using the following formula: $\gamma_{\mathrm{cc}}=-\gamma_{\mathrm{cm}1}\cos\left(\theta_{2}\right)-\gamma_{\mathrm{cm}2}\cos\left(\theta_{1}\right)$ [228].

Various models enable MPA to characterize cell mechanical properties accurately. A simple model given by Theret *et al*. considers the samples with incompressible and homogeneous linear-elastic properties [229]. The influence of micropipette radius was taken into account by Zhou *et al*. and resulted in the neo-Hookean solid model [230]. For viscoelastic properties, a time-dependent model considers the kinetic aspects of cell deformation [231]. Note that the models mentioned here assume a cell as a homogeneous entity, but in fact a cell organizes its components unevenly and enjoys heterogeneous and anisotropic mechanical properties. Since the critical part of precise measurement depends on accurate mechanobiological models, new models have been devised including diffusion models [232], actomyosin cortex models [114], poroelastic models [233] and liquid/cortex models [115, 234]. Beyond that, researchers have designed both one- and two-micropipette systems, which allow multiple manipulation of a single cell, or multiple membranes, simultaneously [112]. The two-micropipette system allows to study cell–cell interactions or ligand–receptor interactions close to their natural state. Low throughput and force resolution are the key limitations of MPA application in cell mechanics. However, the updates in system design have led to increased throughput to 1000 cells per hour instead of 20 cells per hour [235, 236].

MPA, either alone or combined with other experimental modalities, has significantly advanced our understanding of cell mechanics and mechanotransduction. When used alongside 3D confocal microscopy, MPA has revealed that CD4^+^ T-cell deformation is primarily influenced by the relative size of the nucleus rather than by cytoskeletal or other geometric factors [237]. This technique has also been employed by Hong *et al*. to study the bonding properties of TCR-pMHC-CD8, demonstrating that the TCR mechanotransduction signaling loop functions differently in the thymus [238]. During the negative selection of T cells, thymocytes engage TCR and the co-receptor CD8, forming TCR-pMHC-CD8 trimolecular catch bonds, while TCR-pMHC and pMHC-CD8 develop bimolecular slip bonds. The force-induced trimolecular catch bonds, termed ‘dynamic catch’, increase the dwell time between thymocytes and T cells, sustaining TCR signaling and ultimately leading to clonal deletion, a key step in negative selection distinct from positive selection in the thymus. Additionally, Robinson’s study indicated that forces within the F-actin and myosin II systems are shared and mediated by various actin crosslinkers [239]. MPA, when combined with theoretical multi-scale modeling, allows researchers to explore cortical mechanosensing at different scales, revealing that myosin and α-actinin respond to dilation while filamin primarily reacts to shear forces. Using MPA, Wang *et al*. investigated the adhesion forces of progenitor ECs (EPCs) and found these forces to be stronger than those in mature ECs across various matrices, with EPCs demonstrating the strongest bonding strength with anti-CD133 [240] with the recent advances in microfluidic and optical technologies have expanded MPA’s applications. For instance, hydrodynamic micropipette systems integrated with microfluidic devices achieve throughputs of ∼1000 cells per hour [241], while combined setups with phase-modulated surface acoustic waves enable simultaneous measurement of cellular compressibility and Young’s modulus [242]. The continued application of MPA is expected to facilitate further insights into cell force transmission and mechanotransduction in the future.

### Micropipette force sensor

The setup requires a glass micropipette, which must be calibrated for stiffness before use, along with a microscope to monitor the micropipette’s displacement and, in some cases, the cell shape. A micromanipulator accurately positions the tip of the micropipette or microneedle, which is then linked to the cell or molecule of interest [118]. The approach-and-retraction process is repeated multiple times to record deflection and rupture force, providing insights into adhesion dynamics, elasticity, and membrane tension over time [119]. Micropipette force sensor (MFS) can achieve a force resolution as low as 1 pN and a spatial resolution of 1 nm [149].

MFS has been a pioneer in studying cell mechanics since the early 20th century. In 1960, Yoneda investigated the force exerted by a single cilium of *Mytilus edulis*, finding a mean torque of 3.9 × 10^−7 ^dyne·cm, with some variation between 2 and 8 × 10^−7 ^dyne·cm [244]. Kishino later employed microneedles to assess the tensile strength of a single actin filament interacting with myosin motors, measuring around 0.2 pN, while the tensile strength of F-actin was slightly below 100 pN [148]. The binding force of kinesin to microtubules was measured at 5.4 pN [149]. For measuring adhesion and membrane tension of individual vesicles or cells, Colbert *et al*. conducted experiments where cells were mounted at the end of a micropipette attached to a micromanipulator. They analyzed adhesion energy and membrane tension through direct recordings of micropipette deflection [119]. Recently, Shimamoto *et al*. combined MFS with confocal microscopy to study the micromechanics of the metaphase spindle, analyzing its viscoelastic properties over timescales ranging from sub-seconds to minutes [118]. Using the micropipette force spectroscopy technique, Kreis *et al*. measured the adhesion forces of light-sensitive microalgae to surfaces, recording adhesion forces in the range of 1–10 pN [117]. Additionally, MFS was employed to analyze the bending properties of *C. elegans* [246]. In the future, the application of MFS is expected to continually favor cell force transmission studies.

### Fluidic system

The fluidic system discussed here primarily refers to the parallel-plate flow chamber (PPFC) and the miniaturized microfluidic system (Figure 5H). A typical PPFC consists of a polycarbonate distributor, a silicon gasket, and a glass coverslip [247]. The height of the flow path is determined by the thickness of the gasket. Based on Poiseuille flow between parallel plates, the mean flow shear stress (FSS), which approximates wall shear stress (τ), is described as follows:

$$\tau =\frac{6Qµ}{h^{2}w}[248]$$

The PPFC has been widely used to study endothelial exposure to shear stress, as blood flow patterns and shear stress significantly influence the physiological responses of ECs. Ajami *et al*. utilized the PPFC to expose ECs to pulsatile shear and oscillatory shear over various intervals, measuring the transcriptome after exposure [249]. Their transcriptional analysis identified three key sets of responses: cell cycle, oxidative stress and inflammation. This work provides valuable insights into EC function in response to different shear conditions, with critical implications for the pathogenesis of vascular diseases. Schwartz *et al*. investigated the anti-inflammatory effects of high laminar shear stress (FSS) on ECs. They treated ECs with TGFβ2, interleukin 1β (IL-1β), and tumor necrosis factor α (TNFα) under high FSS conditions in a PPFC and quantified the levels of Smad2/3 and other relevant gene expressions. Combined with animal experiments, their findings demonstrated that high FSS dampens Smad2/3 activation atherosclerosis (AS) [250]. Fluidic systems have also been employed to analyze cell adhesion [251], cytokine secretion [124, 125], high-throughput screening, detection and mechanistic study of drugs [121–123]. Zheng *et al*. bridged the gap between cell culture and animal experiments in AS research by creating an early AS model embedded on a microfluidic chip [252]. This design successfully demonstrated that the anti-AS drug probucol exhibited cytotoxicity consistent with clinical findings, which was not observed *in vitro*. They also evaluated the anti-AS efficacy of platinum nanoparticles on the chip, aligning well with *in vivo* results in mice. Fluidic systems are valuable for studying shear stress and its effects on cell responses to various treatments, particularly in blood and urine, thereby mimicking *in vivo* conditions more accurately. The continued use of PPFCs and microfluidic chips enhances research in cell mechanics and life sciences.

### Real-time deformability cytometry

**Figure 10.**
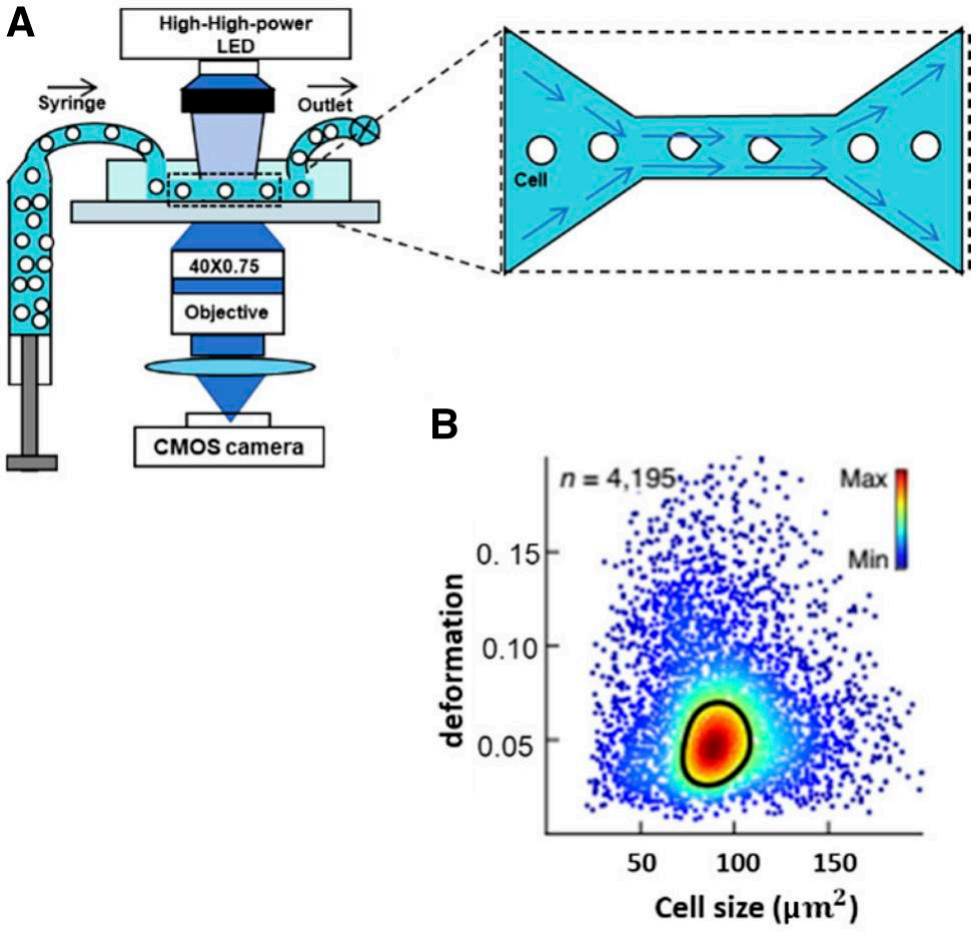
Real-time deformability cytometry (RT-DC). (**A**) Setup and measurement principle. (**B**) Scatter plot of deformation versus cell size. Color indicates a linear density scale; black line, 50%-density contour.

Real-time deformability cytometry (RT-DC) combines microfluidics and flow cytometry to effectively characterize cell mechanics (Figure 10). In an RT-DC setup, cells are flowed through a microfluidic channel constriction, where they deform under shear stresses and pressure gradients without direct contact [126]. Illuminated by a pulsed, high-power LED, cell deformation is recorded by a high-speed complementary metal-oxide semiconductor camera at 2000–4000 frames per second. The deformation (*D*) is calculated as:

$$D\;=\;1-\mathrm{circularity}\;=1-\frac{2\sqrt{\pi \;\mathrm{Area}}}{\mathrm{Perimeter}}[127].$$

A scatter plot is generated with *D* and cell size (cross-sectional area), allowing extraction of the mechanophenotype from the cell population.

To ensure detectable deformation, the microfluidic channel diameter should be designed to accommodate cells that cover 30–90% of the channel width [126, 127]. Image processing yields additional metrics, such as inertia ratio, area ratio, symmetry ratio, differential deformation, and elastic modulus. Cell elasticity modeling under hydrodynamic shear stresses is based on linear elasticity theory and the Stokes equation.

RT-DC has been shown to track the deformation of HL60 cells in response to increasing cytochalasin D concentrations, and to differentiate G2- from M-phase cells during cell cycling. It has also been used to characterize the differentiation of HL60 cells into granulocytes, monocytes, and macrophages. Cytomechanical analysis of human blood revealed three distinct subpopulations—platelets, peripheral blood mononuclear cells and granulocytes—demonstrating its potential for clinical applications [253]. RT-DC also enables comprehensive cell rheological—measurements [254].

An advanced version of RT-DC, called real-time fluorescence and deformability cytometry (RT-FDC), integrates fluorescence imaging with deformability analysis, enabling the examination of cells labeled with fluorescent antibodies or proteins. For example, RT-FDC has been used to analyze mitosis in H2B–mCherry-labeled HeLa cells [253]. Another innovation in RT-DC involves the use of polyethylene oxide in the medium buffer to generate viscoelastic fluids, eliminating the need for sheath fluids. This method also supports high-throughput analysis (10 000–100 000 cells/s) through parallelized channels [255]. RT-DC is particularly suited for measuring the deformation of non-adherent cells. For adherent cells, detachment is required, and the disassembly of FA may alter the cell’s mechanical state. Furthermore, numerical simulations and modeling may not fully capture the structural complexity of cells, as they are often treated as homogeneous, isotropic elastic bodies in these models [256].

### Super-resolution microscope

Super-resolution techniques are invaluable for studying cell biomechanics, providing high-resolution images of cellular substructures and detailed maps of macromolecular assemblies within living cells. These methods enable researchers to probe the mechanical properties and behaviors of cells with unprecedented resolution, significantly advancing our understanding of cellular functions and mechanobiology.

Techniques such as STED (Stimulated Emission Depletion) microscopy, SIM (Structured Illumination Microscopy) and PALM/STORM (Photoactivated Localization Microscopy/Stochastic Optical Reconstruction Microscopy) allow visualization of the cytoskeleton with nanometer precision. For instance, DNA-PAINT (DNA Points Accumulation for Imaging in Nanoscale Topography) has revealed that integrin receptor clustering follows a non-random organization, with complexes spaced 20–30 nm apart [128]. Additionally, STORM has demonstrated that T-lymphocyte activation leads to chromatin decondensation, disruption of the nuclear envelope, and the release of DNA into the cytoplasm [129].

Despite the remarkable detail provided by super-resolution imaging, challenges remain, including phototoxicity, lengthy acquisition times, and the need for sophisticated data processing. Future advancements may focus on combining super-resolution techniques with live-cell imaging and integrating them with complementary methods, such as AFM and TFM, to achieve a more holistic view of cell biomechanics. Indeed, TFM paired with super-resolution microscopy has revealed that filopodia align with the force fields generated by FA [155].

### Fluorescent sensors

**Figure 11.**
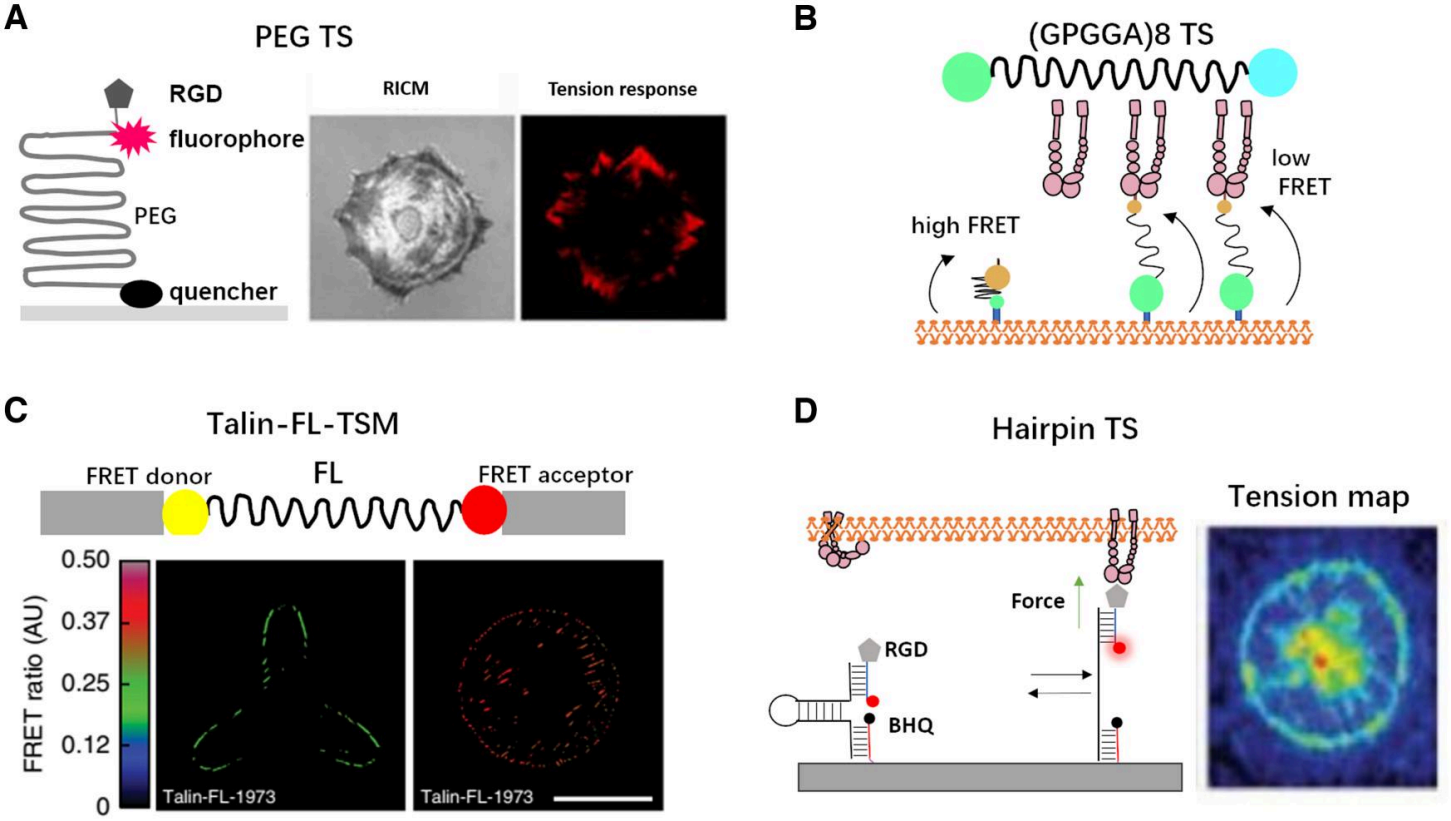
Digital reversible tension sensor (DTS). (**A**) PEG-based TS, adjusting the number of PEG units in the elastic polymer [258, 259]. Different forces yielded FRET index is calibrated, and then cell membrane receptor tensions are investigated. (**B**) (GPGGA)_8_-based TS, elastic repeat GPGGA from silk protein is recruited and expressed in *E. coli* with RGD motif for binding integrin. Further modifications are added for quencher-fluorophore according to click chemistry reaction [260]. (**C**) Talin-based TS (Talin- FL-TSM (tension sensor modules)), which can be used in the study of force transduction. Paired FRET fluorescent proteins were inserted inside of talin molecule after expression in mammalian cells [261]. Here FL mainly unfolds at 3–5 pN at a pulling speed of 500 nm/s. (**D**) Hairpin DNA TS linked with RGD for integrin binding and modified with FRET pairs, is very useful to measure real-time signal for integrin tension. The lower limit reaches up to 4.7 pN [161]. Redrafted from the following articles [128, 258, 269–279]. We acknowledge permission from Springer Nature and ACS.

The instruments mentioned above have significantly advanced the study of cell mechanics. However, to achieve better resolution in measuring cell adhesive forces, practical advancements in force-to-fluorescence conversion have emerged. Researchers have drawn from well-studied biomolecules to create single-molecule tension sensors, using frameworks such as PEG, DNA, peptide nucleic acid (PNA) [257], and peptides, etc. (Figure 11). By conjugating these sensors with target ligands and immobilizing them on a glass substrate, receptors on the cell membrane (such as integrins, TCR and cadherins) can bind to their respective ligands and release a detectable fluorescent signal when the pulling force exceeds the threshold necessary to maintain the folded structure of the sensor. The initial step involves calibrating the mechanical properties of the selected sensor, making instruments like AFM, OT and MT crucial for directly measuring single-molecule unfolding or rupture forces. Fluorophore pairs, such as mTFP1-mCitrine, which exhibit Förster resonance energy transfer (FRET) [267] or quencher-fluorophore pair [268] are designed into the tension sensor according to different needs and are incorporated into the tension sensors based on specific needs. It is important to note that calibration is often performed under constant force or constant pulling speed, leading to discrepancies between the experimentally applied force and the dynamics of living cells. Additionally, the rupture force recorded by tension sensors may vary with cell movement; for example, increasing the pulling time can result in a decrease in rupture force for a given probe. For instance, if the pulling time increases, the rupture force for a given probe will decrease consequently. The following section will summarize the use of tension sensors, which have extensively contributed to investigations of cell mechanics and physiology, categorized according to the information provided by the probes (see Table 3).

**Table 3.** Fluorescent tension sensors used in cell mechanical studies

	Types	Calibration	Detection range (pN)	2D Spatial resolution (μm)	Suitable for cell type	References
DTS (digital tension sensor)	PEG-based	Constant pulling speed mode/AFM	0–20	<1	T-cell adherent cell	[258, 269–271, 287]
	(GPGGA)_8_-based	Constant force mode/OT	1–5	<1	Adherent cell	[10, 260, 272–276]
	DNA hairpin	Constant pulling speed mode/OT	4.7–19.3	<1	T cell	[128, 132, 161, 277–279]
	FL-based	Constant pulling speed/OT	3–5	<1	Adherent cell	[261, 288]
BTS (binary tension sensor)	TGT	Constant force mode/MT	12–54	<1	Adherent cell	[263, 283, 284]
	ITS	Constant force mode/MT	12–54	<1	Platelet, adherent cell	[251, 262, 285, 286]
	Nano yoyo	Constant pulling speed mode/OT	>4	<1	Adherent cell	[265]
	YFP-based	Constant pulling speed mode/AFM	>97 ± 27.1	<1	Fibroblast, adherent cell	[264]
	I27-based	Constant pulling speed mode/AFM	>80	<1	Adherent cell	[131, 266, 289]
CTS (combo tension sensors)	RSDTP	Constant loading rate mode/MT	4–60	<1	Adherent cell	[290]
Membrane tension sensor	FLiptR	–	–	–	Adherent and suspended cell	[291]
	(GPGGA)_8_-based	Constant force mode/OT	1–5	–	Adherent and suspended cell	[162]

#### Digital reversible tension sensor

Digital reversible tension sensor (DTS) has the advantage of reporting the force level as well as distance change between dye-dye or dye-quencher pairs. Therefore, it is expected to supply more details during cell experiments. This design applies to PEG-, hairpin DNA-based, and (GPGGA)_8_-, ferredoxin-like peptide (FL)-based tension sensors (Figure 11) [128, 132, 161, 258, 260, 269–279]. In most cases, these tension sensors could refold upon relaxation and thus are reversible. For DNA TS, the force *F*_1/2_ (the average dwell times in the folded and unfolded states are equal) is calibrated using the equation below:

$$F_{1/2}=(\Delta G_{\text{fold}}+\Delta G_{\text{stretch}})/\Delta x[132]$$

where ΔG_fold_ is the free energy of unfolding the hairpin at *F = 0,* ΔG_stretch_ is the free energy of stretching the ssDNA from *F = *0 to *F = F*_1/2_.

DTS can be designed using non-biological polymers such as PEG, allowing for adjustable tension sensitivity based on the PEG length or the number of repeating units. For instance, Salaita selected PEG12, PEG24 and PEG75, with PEG24 expected to unfold within a force range of 0–20 pN, where >95% of the maximum fluorescence intensity is observed at 20 pN pulling force [258]. The Salaita Group first calibrated the mechanical force between epithelial growth factor and its receptor using PEG tension sensors, demonstrating that mechanical forces are linked to epithelial growth factor (EGF) internalization. They later developed a hairpin DNA-based tension sensor, with sensitivity in the few pN range, by manipulating GC content, allowing them to investigate force dynamics in TCR-pMHC interactions [280] and the mechanical activation of integrin αIIbβ3 [281]. The direction of force has since been considered a critical factor in force analysis, leading to the application of these tension sensors in studies of platelets and other cells [282]. Elastic peptide FL and (GPGGA)_8_ have the advantage of being expressed in the cell, and thus could be expanded into the force transmission study from the outside to the inside of cells. FL has an unfolding force mainly at the around 3–5 pN, while most (GPGGA)_8_ may be unfolded at 1–5 pN. A FRET pair consisting of Ypet-FL-mCherry was genetically inserted into talin, creating tension sensor modules (FL-TSM). This setup confirmed tension in talin during cell adhesion, revealing that the talin head and rod domains experience forces exceeding 7 pN, while forces >3 pN are observed between the C-terminal F-actin-binding sites [261]. The mTFP-(GPGGA)_8_-venus vinculin tension sensor was constructed to calibrate tension across vinculin in stable FAs, measuring approximately 2.5 pN. This low force in vinculin is associated with disassembly or sliding of FAs at the trailing edge of migrating cells [10]. The DTS technology is poised to facilitate new discoveries in cell mechanics and relies heavily on advanced fluorescence microscopy techniques.

#### Binary tension sensor

**Figure 12.**
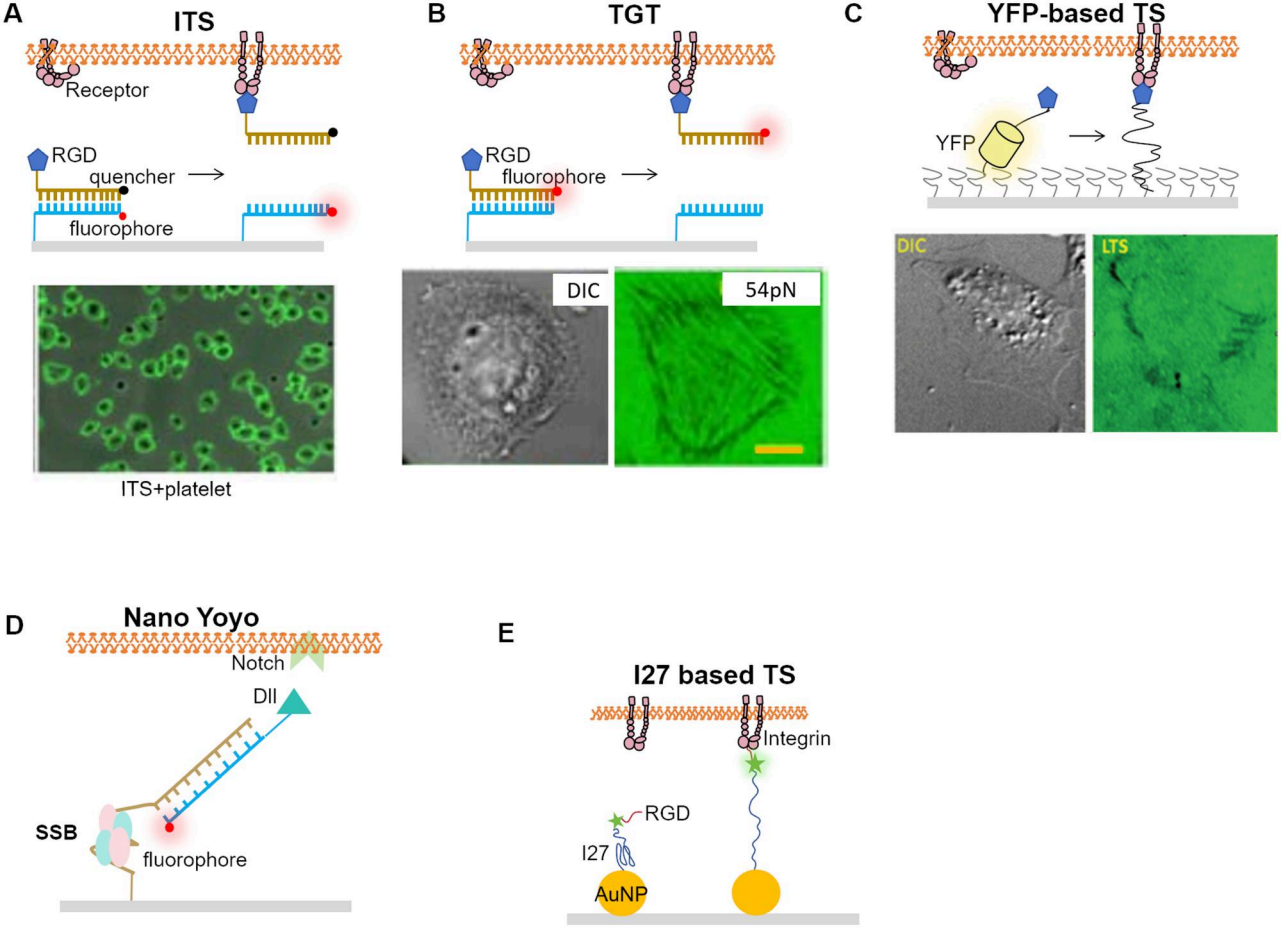
Binary tension sensors. (**A**) ITS, conjugating quencher-fluorophore pairs into TGT module, and thus providing a better signal-to-noise ratio [262]. Integrin tensions of activated platelets are examined with high throughput. (**B**) TGT, a dsDNA fragment conjugated with RGD ligand where the force signal is detected as long as the fluorescence is lost [263]. (**C**) YFP-based TS, engineered with RGD coding sequence and transfected into bacteria, helps map integrin tensions without additional chemical modification [264]. (**D**) Nano YoYo, an elegant design inspired from SSB–ssDNA interaction, acquires sensitivity up to 4 pN [265]. (**E**) I27-based TS, linked with RGD and green fluorescence protein (GFP), can be unfolded at high force and reports cell traction forces by integrin [266]. Redrafted from the following articles [229, 232, 235–237]. We acknowledge permission from Springer Nature, RSC, ACS, Wiley and AA.

Binary tension sensors (BTS) primarily refer to double-stranded DNA (dsDNA)-based tension sensors (Figure 12). The force threshold for rupturing dsDNA establishes the sensor’s capability and resolution in measuring force levels, with a typical range between 10 and 60 pN. Taking the tension gauge tether (TGT) for an example, the critical force *fc*
 

$$fc=2f_{1}\chi^{-1}\text{tanh}\left(\chi N/2\right)[130]$$

where *f_1_* is the force required to rupture a single base pair, χ =$\sqrt{2R/Q}$ is a function of the spring constant (Q) between neighbors in a strand, and the spring constant (*R*) between base pairs, *N* is the DNA base pair number.

The Wang Lab has developed two types of BTS: TGT [263, 283, 284] and integrative tension sensor (ITS) [251, 262, 285, 286] (Figure 12A, B). One of the key advantages of these sensors is their programmability, allowing for continuous or high- resolution adjustments to the probe rupture threshold under optimal conditions. The TGT has been used to measure the tension levels associated with integrin-mediated cell adhesion, revealing that a minimum force of 43 pN is required. For cell spreading, a higher tension of over 54 pN is necessary for the formation of mature FA The ITS complements the TGT by incorporating a quencher-fluorophore pair, which enhances the signal-to-noise ratio. Using the ITS, researchers have mapped the forces mediated by integrin αIIbβ3 in platelets, detecting two distinct force levels during platelet adhesion and contraction [251]. To counteract the degradation of dsDNA-based tension sensors in the presence of nucleases in FAs or podosomes, the anti-nuclease ITS was developed, replacing one DNA strand with a PNA chain. This sensor has been employed to investigate force dynamics in cancer cells such as HeLa and Medullary thyroid carcinoma (MTC) cells [257]. Additionally, a label-free tension sensor has been created by fusing the fluorescent protein YFP to an RGD peptide, allowing it to function as a tension sensor without external labels [264] (Figure 11C). While this YFP tension sensor is both label-free and nuclease-resistant, it necessitates a significantly higher pulling force (>97 pN) to produce a detectable signal. Another two are Nano yoyo [265] and I27-based sensors [131, 266] (Figure 12D, E). The Nano yoyo utilizes the inherent properties of *E. coli* single-stranded DNA-binding protein (SSB) and single-stranded DNA (ssDNA), demonstrating a low rupture force (∼4 pN at a pulling speed of 500 nm/s) [31]. With these tension sensors, the Notch signaling pathway was found to be activated below 12 pN at first by TGT, and then was narrowed down at the range of 4–12 pN by Nano yoyo. Furthermore, research has shown that platelet activation may depend on integrin tension exceeding 54 pN [281].

#### Combo tension sensor

**Figure 13.**
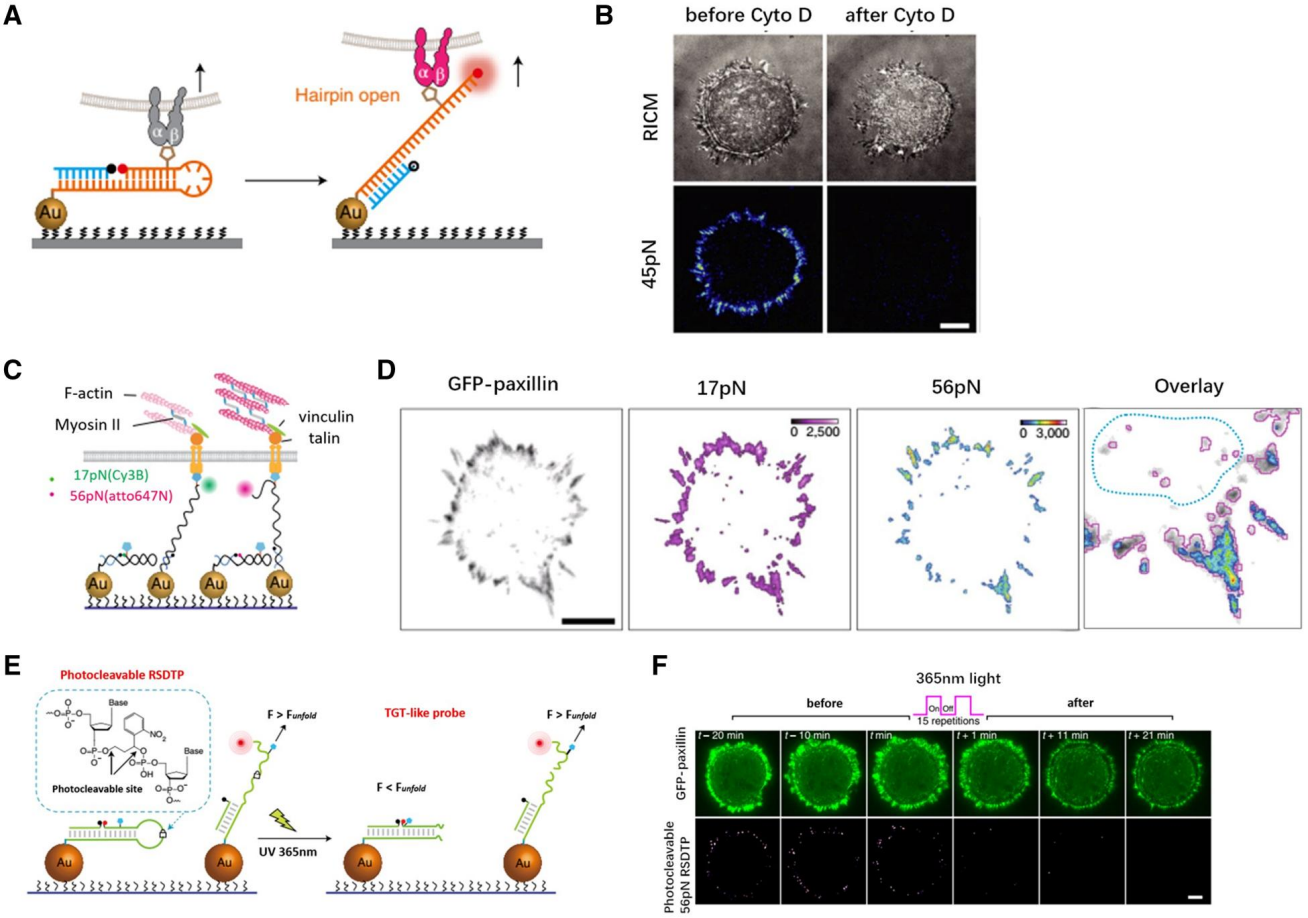
Combo tension sensors. (**A**) The design of RSDTP (reversible shearing DNA-based tension probe), which adapts from ITS and hairpin TS. The hairpin structure makes it reversible between folding and unfolding states, while ITS moiety imbues the upper threshold of rupturing RSDTP. Altogether RSDTP has a broad measuring range of between 4 and 60 pN [290]. (**B**) Representative images of reflection interference contrast microscopy (RICM) and integrin-mediated tension of a cell before and after treated with cytochalasin D. (**C**) Schematic of real-time force images with multiplexed RSDTPs. (**D**) Representative total internal reflection fluorescence (TIRF) images of GFP–paxillin, tension signals of 17 pN (Cy3B) and 56 pN (Atto647N) and overlay of all channels. (**E**) Schematic of photocleavable RSDTP. A photocleavable linker and a non-nucleotide moiety were incorporated into the sugar-phosphate backbone within the loop region, linking two nucleotide sequences through a short, UV-photocleavable C3 spacer arm. (**F**) Upper: schematic of the periodic pulse of UV illumination. Lower: time-lapse TIRF images of GFP–paxillin and single-molecule tension signals of a GFP–paxillin-expressing cell adhered to a mixed-sensor surface (2% 56-pN photocleavable RSDTP and 98% 56-pN TGT) show dynamic changes of FAs before and after photocleavage. Scale bar: 10 μm. We acknowledge permission from the Copyright Clearance Center of the Springer Nature to reuse the article [290].

The Liu Lab developed a tension sensor known as the reversible shearing DNA-based tension probe (RSDTP), which combines the advantages of hairpin DNA hybridization and dsDNA structures (Figure 13). The RSDTP probes molecular forces in the pN range, specifically between 4 and 60 pN, transmitted by cells. The hairpin DNA component allows for reversible reactions (Figure 13B), while the shear mode TGT expands the force range up to 60 pN. Importantly, the TGT modulates the upper limit of the probe by adjusting the tether position of RGD, addressing concerns that irreversible TGT might disrupt normal cell physiology during experiments [290]. It proved that mechanically strong integrins maintain the FA architecture. By incorporating a photocleavable spacer (which converts RSDTP into TGT upon UV exposure), they mixed 2% 56-pN photocleavable RSDTP with 98% 56-pN TGT. They observed that the lifetime of FAs decreased after photocleavage, indicating that FA stability is influenced by a small number of integrins experiencing higher tensions. Further calibration revealed that the forces exerted by integrins at nascent FAs are approximately 45 pN, explaining why the 40 pN TGT supports cell adhesion, while stable adhesion requires higher tension. Photobleaching experiments estimated that there are around seven integrin molecules per nanocluster (nascent FA) with a peak force of 45 pN. This probe could switch from a reversible to an irreversible state by UV light after being engineered with a photocleavable bond, which expanding its application in studying cell mechanics.

#### Membrane tension sensor

**Figure 14.**
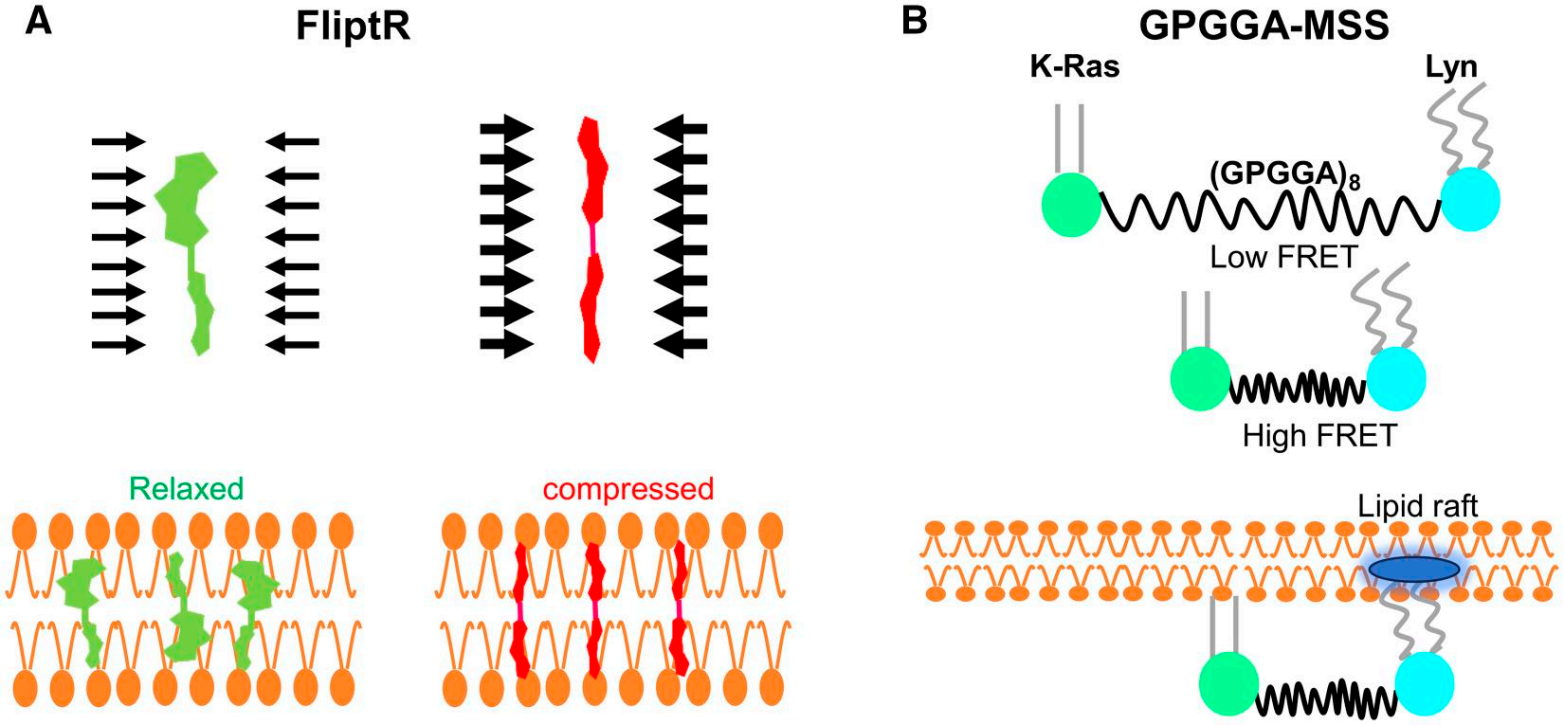
Membrane tension sensors. (**A**) Cell membrane tension sensor FliptR (fluorescent lipid tension reporter). The FliptR can be used to monitor cell membrane tension since the FliptR molecule changes its optical property under lateral pressure from cell membrane [291]. (**B**) The engineered protein membrane TS based on (GPGGA)_8_. Membrane shear stress sensor (GPGGA)_8_-MSS, in which two membrane-binding proteins lyn and K-Ras were engineered at the two ends. MSS and K-MSS expressed cells are treated with sucrose and then imaged [162]. ECFP, cyan fluorescence protein; KMSS is a head-less mutant, the control probe; MSS, membrane-bound FRET-based tension sensor; YPet, yellow fluorescent protein for energy transfer. Redrafted from the following articles [162, 291]. We acknowledge permission from Springer Nature, and Cell Press.

Membrane tension is closely linked to various cellular activities, with increased tension observed during cell migration and division. Changes in cell volume often correlate with fluctuations in membrane tension, impacting processes such as endocytosis, phagocytosis, and cell spreading. As a result, several methods have been developed to calibrate membrane tension to better understand its relationship with cell activities. One approach involves extracting small membrane tubes from the plasma membrane; however, the accuracy of these measurements is questionable due to the difficulty in deconvoluting the interaction between the cell membrane and the actin cortex [292]. A novel tool called the fluorescent lipid tension reporter (FliptR) responds to membrane tension changes in living cells. FliptR contains two large dithienothiophene flippers that twist due to repulsion between methyl groups and endocyclic sulfurs near a rotatable bond. A negatively charged carboxylate facilitates proper membrane insertion (Figure 14A) [291]. Using fluorescence lifetime imaging microscopy, FliptR quantifies membrane tension by measuring changes in planarization in response to increased cell membrane pressure. This probe can also detect alterations in the organization of lipid bilayer membranes.

As an indirect measurement of membrane tension, FliptR may be sensitive to changes in membrane composition or phase separation. Another design for measuring shear stress in cell membranes is the ECFP-(GPGGA)_8_-YPet, known as the membrane-bound FRET-based tension sensor (MSS) (Figure 14B) [162]. MSS links to two membrane-binding proteins, Lyn in lipid rafts and K-Ras in non-lipid rafts. An increase in membrane tension correlates with a lower FRET ratio. The elastic (GPGGA)_8_ segment determines the sensor’s sensitivity, which ranges from 1 to 5 pN. Using MSS, Wang *et al*. reported a positive correlation between cell membrane tension and membrane fluidity, along with a detectable heterogeneous distribution of tension under shear stress. Under low shear stress, embrane tension increased due to fewer stress fibers in the cytoskeleton, which reduced restrictions on lipid mobility, while lipid rafts were constrained by microtubules. Overall, the measurement of membrane tension still requires more effective tools, representing a promising direction in cell mechanics and mechanotransduction research.

## Conclusions and perspectives

This review begins by providing a background on cell force sensing, covering the molecular components involved, associated biochemical signaling pathways, and the significance of cell force sensing for proper cellular function. We then summarize various analytical methods for quantifying cellular mechanics, including TFM, µFSA, OT, MT, AFM, fluidic systems, MPA and fluorescent probes. While these advanced techniques have significantly enhanced research in cell force sensing, each method has systemic limitations when it comes to studying cell mechanics. For example, TFM requires substantial computational resources, while OT, MT and AFM demonstrate low efficiency for multiple measurements over short timescales. Additionally, membrane tension sensors often lack the sensitivity needed for precise tension measurement. Lastly, while TGT, ITS and hairpin tension sensors are effective for visualization, they fall short in time resolution.

Understanding cell force sensing is critical for cellular function, making it imperative to uncover the mechanisms behind cellular mechanical responses. Future development of novel sensors, enhancements to existing instruments, and the integration of various platforms require ongoing support. The following sections outline our recommendations for future studies in cell mechanics.

### Updating sensors

Cell force sensors have been developed using various materials, including elastic peptides, dsDNA, and polymers like FL, (GPGAA)_8_, TGT, ITS and PEG. These tension sensors enable the measurement of cell forces ranging from a few pN up to 60 pN. Through these methods, the Salaita Group has identified the mechanical thresholds for integrin activation in platelets and T cells [132, 281]. However, in cancer cells, FAs often contain nucleases that degrade dsDNA, making TGT or ITS unsuitable for force calibration in these contexts. To address this, an updated version of ITS incorporating PNA was designed, allowing for the investigation of integrin tensions in podosomes [293]. Some sensors have also been designed to quantify both the direction and magnitude of forces exerted by cells on glass surfaces [159]. Additionally, a ratiometric tension sensor was invented to monitor integrin tension magnitude, validating the rigidity dependence of integrin tensions [294]. A sensor incorporating DNA origami, specifically a novel library of six-helix-bundle DNA-origami tension probes, was engineered with tunable tension-reporting hairpins (each with a distinct tension response threshold) and a variable number of cell-receptor ligands. This innovation opens new possibilities for studying and regulating biophysical processes involving cooperativity and multivalency [281]. Despite these advances, current tension sensors are typically limited to monitor cell force sensing over a few hours, which limits ability to check the cell performance through differentiation or development stages which occur over days to months. Consequently, a key question remains:

how can we design a tension sensor that is suitable for monitoring tissue development over extended periods?

### Paying attention to real-time interactions between receptor and ligand

Current tension sensors are calibrated using advanced techniques such as MT, OT and AFM, typically at constant force or constant pulling speed. However, researchers still lack a definitive understanding of the duration of receptor-ligand interactions during cell–cell crosstalk. For instance, Strohmeyer *et al*. estimated that integrins bind to fibronectin for less than one second during initial cell adhesion, as evidenced by AFM experiments [52]. Unfortunately, this finding has not been followed up with further research. Recently, the loading rates between integrins and their ligands, on the order of a few pN/s, have been examined by three different labs [295–297]. While OT, AFM and MT can detect ligand–receptor interactions with microsecond sensitivity, they are limited to analyzing only small areas of the cell, making it difficult to study functional structures like FAs. We encourage our peers to focus on real-time interactions of ligand–receptor complexes to deepen our understanding of these critical processes.

### Applying artificial intelligence and machine learning for handling force-sensing data

Proficiency in using MATLAB or Python to develop specific codes for analyzing cell force data is essential. Artificial intelligence (AI) and machine learning have already been successfully applied in industries such as aerospace and precision manufacturing, clinical diagnosis and scientific research, etc. [298–300] Therefore, the integration of AI and machine learning for analyzing force-sensing data enables more accurate and efficient interpretation of complex mechanical forces within biological systems. These technologies can process large datasets generated by force-sensing assays, identifying patterns and correlations that might be difficult to discern through traditional analysis methods.

### 3D Tissue force modeling

Studying forces in 3D tissue models or *in vivo* environments is challenging due to the inherent complexity of these systems. While 3D models more accurately replicate tissue architecture and mechanical interactions compared to 2D cultures, measuring forces within them requires advanced techniques capable of capturing multidimensional, dynamic data. For example, TFM can map cell contraction in 3D hydrogels, but its resolution is lower than that achieved on 2D surfaces. Additionally, *in vivo* environments are affected by factors such as tissue heterogeneity, fluid dynamics, and cellular interactions, making it difficult to isolate specific force-related variables. Technical limitations, including spatial resolution and sensor sensitivity, further complicate precise force measurement in these contexts. Addressing these challenges will require innovative solutions in the future.

### Updating platforms and more

Since AFM and OT cannot detect interaction events with high temporal and spatial resolution, and tension sensors can map cell force distributions, there is an opportunity to combine these methods to explore cell mechanics from a new perspective. One attempt has been made to construct a platform integrating tension probes with microfluidic chips [301]. Additionally, mechanosensitive ion channels such as PIEZO-1 and TRPV4 are expected to respond to shear flow, but their detailed mechanisms require further investigation. The adhesion of circulating cancer cells in blood flow also necessitates a more in-depth study. It is anticipated that fluidic and microfluidic systems will play a crucial role in facilitating these investigations in the future. By functionalizing DNA tension probes on soft hydrogel surfaces and coupling TFM with tension sensors, Liu’s lab developed a valuable approach to investigate cell rigidity sensing and mechanotransduction [302]. Furthermore, Li *et al*. developed photonic crystal cellular force microscopy, a technique capable of detecting vertical cell forces with high speed [303].
